# A Comprehensive review of data-driven approaches for forecasting production from unconventional reservoirs: best practices and future directions

**DOI:** 10.1007/s10462-024-10865-5

**Published:** 2024-07-22

**Authors:** Hamid Rahmanifard, Ian Gates

**Affiliations:** Department of Chemical and Petroleum Engineering, Schulich School of Engineering, University of Calgary, 2500 University Dr. NW, Calgary, AB T2N 1N4 Canada

**Keywords:** Unconventional reservoirs, Machine learning, Data analytics, Artificial intelligence, Hydrocarbon production

## Abstract

Prediction of well production from unconventional reservoirs is a complex problem given an incomplete understanding of physics despite large amounts of data. Recently, Data Analytics Techniques (DAT) have emerged as an effective approach for production forecasting for unconventional reservoirs. In some of these approaches, DAT are combined with physics-based models to capture the essential physical mechanisms of fluid flow in porous media, while leveraging the power of data-driven methods to account for uncertainties and heterogeneities. Here, we provide an overview of the applications and performance of DAT for production forecasting of unconventional reservoirs examining and comparing predictive models using different algorithms, validation benchmarks, input data, number of wells, and formation types. We also discuss the strengths and limitations of each model, as well as the challenges and opportunities for future research in this field. Our analysis shows that machine learning (ML) based models can achieve satisfactory performance in forecasting production from unconventional reservoirs. We measure the performance of the models using two dimensionless metrics: mean absolute percentage error (MAPE) and coefficient of determination (R^2^). The predicted and actual production data show a high degree of agreement, as most of the models have a low error rate and a strong correlation. Specifically, ~ 65% of the models have MAPE less than 20%, and more than 80% of the models have R^2^ higher than 0.6. Therefore, we expect that DAT can improve the reliability and robustness of production forecasting for unconventional resources. However, we also identify some areas for future improvement, such as developing new ML algorithms, combining DAT with physics-based models, and establishing multi-perspective approaches for comparing model performance.

**Supplementary Information:**

The online version contains supplementary material available at 10.1007/s10462-024-10865-5.

## Introduction

**Fig. 1:**
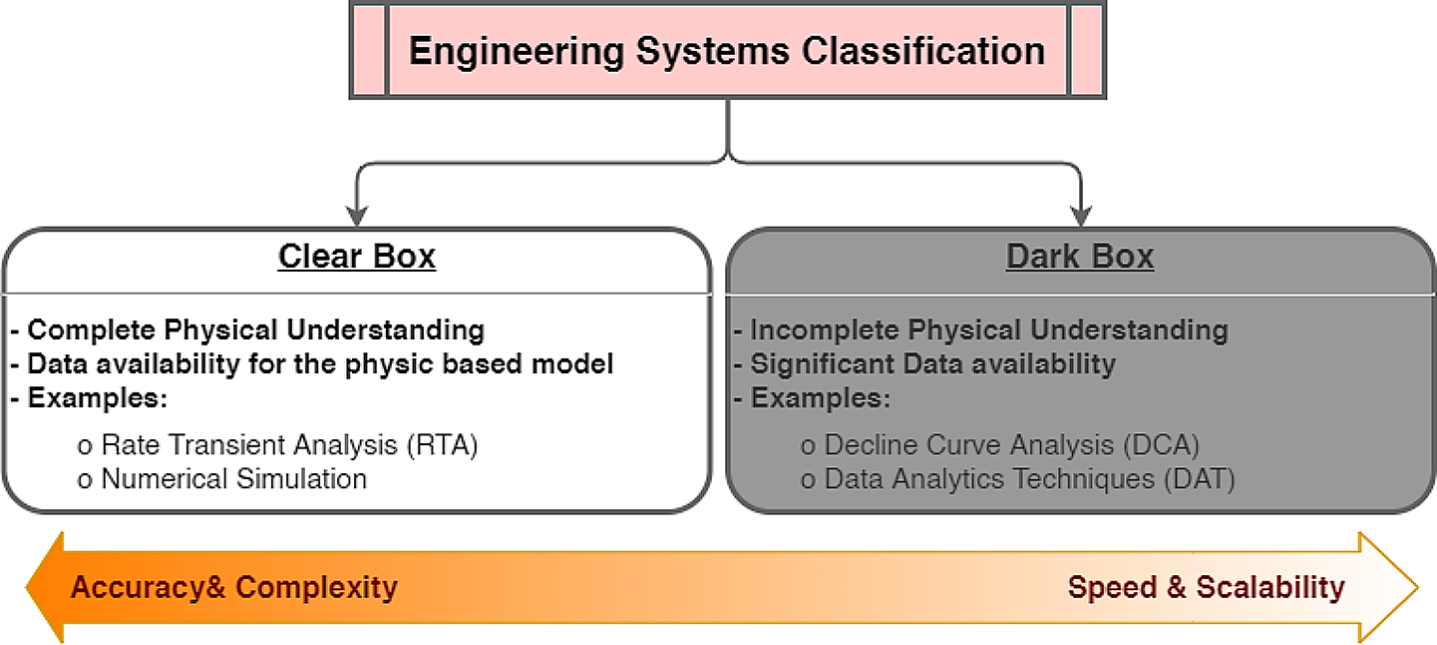
Two-box classification of engineering systems

Hydrocarbon production from unconventional reservoirs requires integrating different technologies, including long lateral horizontal drilling with multi-cluster, multi-stage hydraulic fracture systems that activate natural fracture networks in unconventional formations. The highly permeable fracture system creates a large contact area within the reservoir enabling high flow rate of hydrocarbons to the surface (Mohaghegh 2013; Mohaghegh et al. 2017). Because of massive multi-cluster and multi-stage hydraulic fractures, modelling production from unconventional reservoirs is challenging. As shown in Fig. 1, existing approaches can be divided into two broad categories or ‘boxes’ that can be used for classifying many problems in nature, including hydrocarbon production from unconventional reservoirs (Clarkson 2013; Liu et al. 2021; Mohaghegh 2013; Mohaghegh et al. 2017).

A clear box engineering system is one that we understand the physical mechanisms completely. This requires a full grasp of the physics, physical properties, and a suitable mathematical model to capture them (Liu et al. 2021). Rate Transient Analysis (RTA) and numerical simulations are two examples of physics-based models used for production forecasting in unconventional reservoirs.

Clear box models offer both advantages and limitations in modelling production from unconventional reservoirs. These models provide a detailed understanding of physical mechanisms, capturing essential aspects such as fluid flow, rock properties, and underlying physics. By relying on well-defined assumptions and principles (such as material balance and reservoir engineering concepts), clear box models achieve accuracy under known conditions. Engineers and geoscientists can confidently interpret results based on fundamental physical principles (Liu et al. 2021; Mohaghegh 2013; Mohaghegh et al. 2017). However, these models come with computational intensity, especially in the case of numerical simulations and complex analytical solutions. Additionally, they often require simplifications and assumptions (such as boundary conditions) that may not fully represent real-world complexities. In unconventional reservoirs characterized by heterogeneity, fractures, and complex fluid behavior, clear box models may struggle due to their inherent limitations (Liu et al. 2021; Mohaghegh et al. 2017).

On the other hand, a dark-box system is one that we have a poor understanding of the physical mechanisms with a large amount of data on the system responses to different inputs. In general, dark box approaches are faster and more scalable whereas clear box systems provide more accurate results with greater complexity. Supporting information contains more information about the methods mentioned in the clear and dark box approaches.

Fluid flow in unconventional reservoirs is a complex phenomenon that involves rock and fluid interactions and pore confinement effects on hydrocarbon phase behaviour, which are often not fully understood. Therefore, production forecasting from unconventional reservoirs can be considered a dark-box problem, which requires empirical or data-driven methods to capture the system responses (Liu et al. 2016; Luo et al. 2018; Shabib-Asl et al. 2020). Two common approaches that are used for this purpose are decline curve analysis (DCA) and data analytics techniques (DAT). DCA is a quick and easy method that matches a curve to the historical production data and extends it to predict future production rates and reserves. However, DCA assumes constant conditions, and neglects the effects of well interference or operational changes (Han et al. 2019; Manda and Nkazi 2020; Yehia et al. 2022). DAT are more sophisticated methods that use machine learning (ML) algorithms to learn from the data and make predictions. DAT can deal with complex and nonlinear problems, as well as include various types of data, such as geological, geophysical, petrophysical, and operational data (Mohaghegh 2017; Schuetter et al. 2018). Consequently, a more practical solution to production forecasting from unconventional reservoirs could be based on DAT or the combination of DAT and physics-based models.

Dark box approaches that use DAT have recently gained attention in many scientific and engineering fields (Li et al. 2021a; Liu et al. 2021; Mishra and Lin 2017; Mohaghegh 2013; Noshi et al. 2018; Syed et al. 2021). However, despite more than a decade of applying DAT for production forecasting from unconventional reservoirs, there is no comprehensive and comparative evaluation of the performance of the developed predictive models in the literature. Hence, we aim to critically review the efforts and challenges of using DAT for predicting production from unconventional reservoirs. We also provide insights into the factors that influence the successful application of DAT for production forecasting from unconventional reservoirs. We do not cover physics-based methods, i.e., clear-box approaches, in this review, as they have been extensively studied in previous works (Behmanesh et al. 2018; Clarkson 2013; Duong 2010; Huang et al. 2016, 2017; Ilk et al. 2008; Maulianda et al. 2019; Mohaghegh 2017; Nobakht et al. 2012; Qanbari and Clarkson 2016; Sureshjani and Gerami 2011; Valkó and Lee 2010).

This paper has the following structure: We start by giving a brief overview of the general workflow for using DAT to predict the well performance. Next, we review the literature on the existing DAT methods for production forecasting. Finally, we discuss the performance of the developed predictive models and suggest some future research directions.

## Data analytics techniques (DAT)

Data analytics is the practice of finding key insights and valuable conclusions from a large dataset to support decision-making (Bose 2009; Elgendy and Elragal 2014; Pedamkar 2020). Depending on the objective, data analytics can be classified into three categories (Bose 2009; Pedamkar 2020):

- Descriptive Analytics, which describes past data and characterizes and quantifies the distribution of historical data (e.g., quartiles, median, mode, and mean),
- Predictive Analytics, which uses patterns and trends in past data to build the model, forecast process behaviour, and identify potential risks,
- Prescriptive Analytics, which provides recommendations based on both descriptive and predictive analysis.

**Fig. 2:**
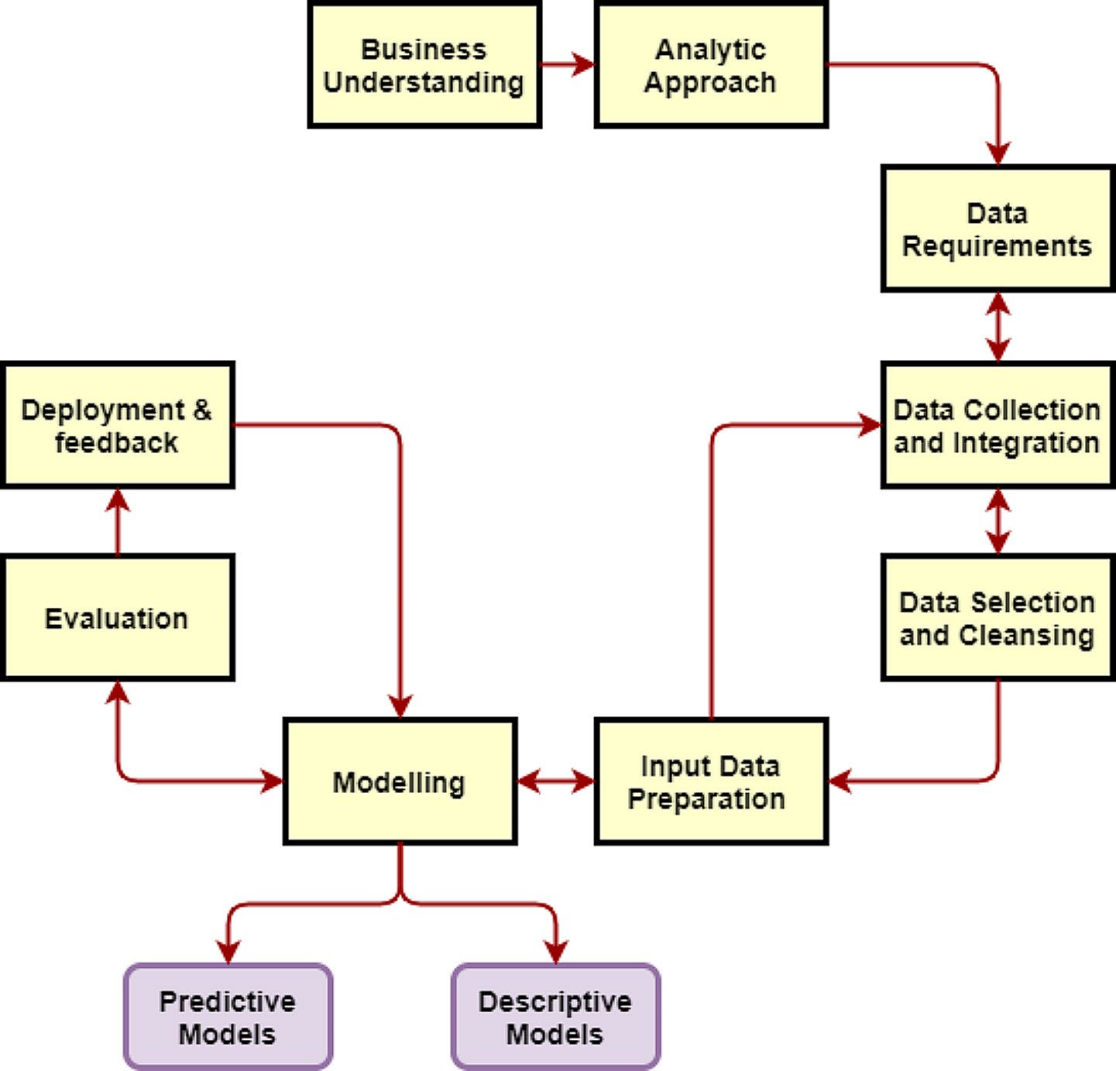
The general workflow to predict well production with DAT.

Figure 2 shows different stages of data processing in data analytics. The first stage is to extract data from various sources and perform cleansing (Descriptive Analytics). In the next stages, valuable insights and conclusions are derived from the cleaned database (Predictive and Prescriptive Analytics).

**Fig. 3:**
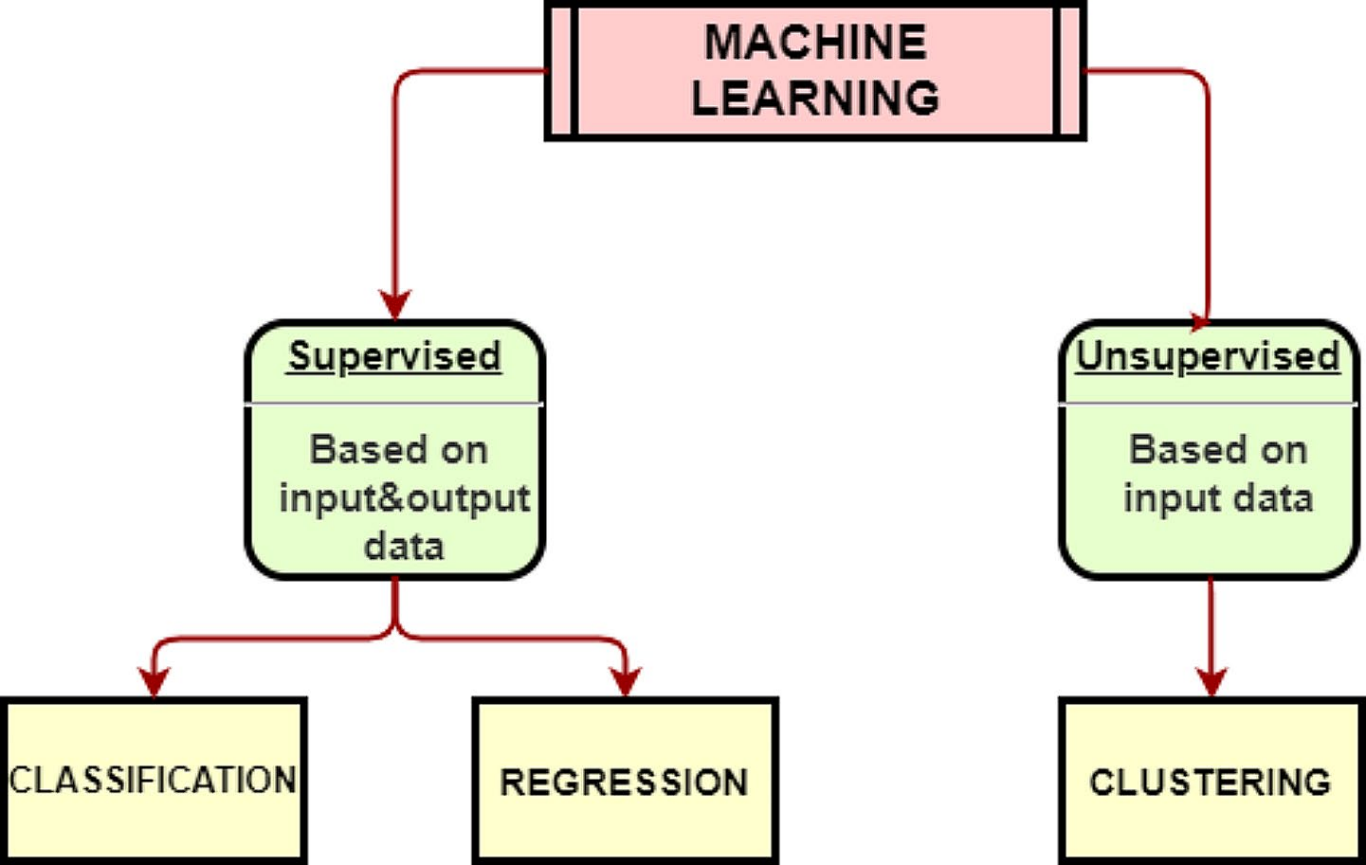
Machine learning algorithms (MathWorks 2020a)

Machine Learning (ML) is a subfield of Artificial Intelligence (AI) that deals with learning from data and making predictions or decisions based on the learned patterns (Mitchell 2010). ML algorithms can be used for both Data Mining and AI tasks, such as exploration, learning, identifying useful patterns, and forecasting the process behaviour. ML algorithms can be classified into two major groups: supervised and unsupervised methods, as shown in Fig. 3,^1^ (Alloghani et al. 2020; Rajoub 2020). Supervised methods use labelled data to train the model and make predictions, while unsupervised methods use unlabeled data to find patterns or structures. Some examples of supervised methods are classification and regression, and some examples of unsupervised methods are clustering and dimensionality reduction.

**Fig. 4:**
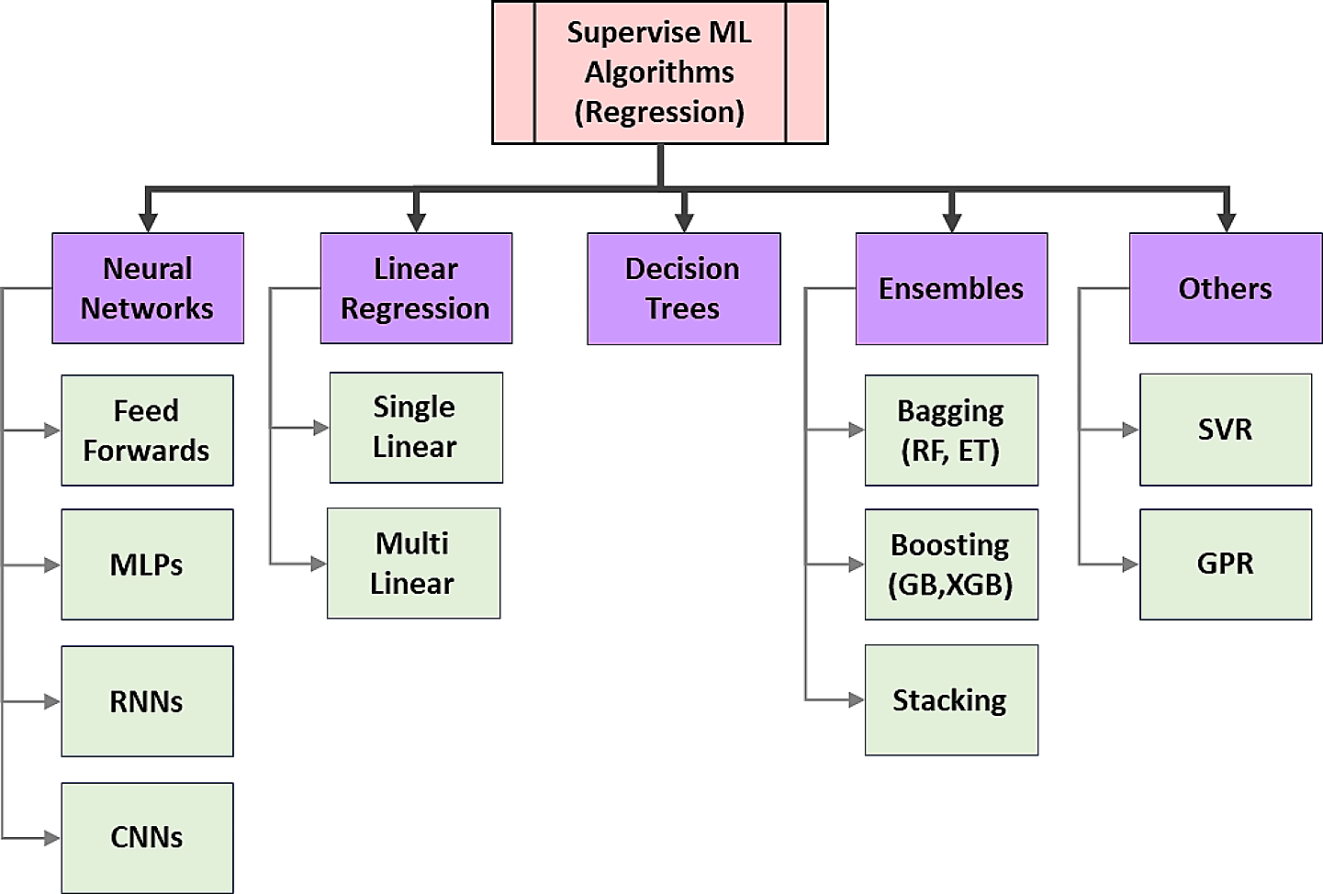
Different ML algorithms for regression purposes (Mathworks 2020b; Glossary 2017; Pedamkar 2020)

The task of production forecasting from unconventional resources falls within the domain of supervised ML regression. In this context, both input and output data are labeled and predominantly consist of numerical variables. Figure 4 illustrates the most commonly used ML algorithms for regression purposes.

Table 1 provides a comprehensive overview of the advantages and limitations associated with these regression methods. Understanding these factors is essential when choosing the most suitable approach for a specific dataset and forecasting scenario. Researchers and practitioners can make informed decisions by comparing algorithms such as Artificial Neural Networks (ANN), Linear Regression (LR), Support Vector Regression (SVR), Classification and Regression Trees (CART), Ensemble methods, Random Forest (RF), and Extreme Gradient Boosting (XGB).

**Table 1:** A comparison of the distinct features of different ML algorithms for regression tasks: ANNs, LR, RF, SVR, CART, XGB, and ensembles (Breiman 2001; Brownlee 2017, 2018; Chen and Guestrin 2016; Deuschle 2018; Dobilas 2020; Ebden 2008; Mathworks 2020b; McElroy et al. 2021; Mitchell 2010; Mohr 2018; Otero et al. 2017; Rahmanifard et al. 2020; Rahmanifard and Plaksina 2019, 2018; scikit-learn 2020; Smola et al. 2004; Zhong et al. 2020)

Algorithm	Advantages	Limitations
ANNs	- Highly adaptable to various types of data and tasks. - Capable of modeling complex, non-linear relationships. - Self-learning capabilities from provided data - Well-suited for time series predictions	- Require a lot of data and computational resources to train. - Prone to overfitting and underfitting if not tuned properly - Difficult to interpret and explain
Linear Regression	- Simple to implement and easy to interpret - Computationally efficient - Model coefficients provide insight into relationships	- Assumes a linear relationship between variables, which may oversimplify real-world problems - Sensitive to outliers - Prone to underfitting complex datasets
SVR	- Can handle outliers, and high-dimensional data - Robust to outliers in the dataset - Appropriate for rapid assessment of reservoir characteristics.	- Require tuning the parameters - Choosing an appropriate kernel function can be challenging - Sensitive to the choice of hyperparameters
CART	- Easy to understand and interpret - Can handle both numerical and categorical data. - Non-parametric, meaning it does not assume any distribution of the data	- Prone to overfitting and high variance - Unstable, as small changes in the data can lead to a different tree - May not perform well on imbalanced data
Ensembles	- Improves prediction accuracy. - Reduces the likelihood of overfitting	- Can be computationally expensive. - More complex to implement than single models - Computationally expensive and slow to train and predict, as they involve multiple models - Require a good choice of base models, meta-models, parameters, or thresholds to avoid underfitting or overfitting.
RF	- Handle nonlinear relationships, missing values, and high-dimensional data - Reduce overfitting and improve generalization	- May lose some information or accuracy due to sampling or averaging - May not perform well on very noisy or sparse data
XGB	- Can handle nonlinear relationships, missing values, and high-dimensional data - Includes regularization which helps in preventing overfitting	- Can be challenging to tune due to the number of hyperparameters - Prone to overfitting if not tuned properly - May not perform well on very noisy or sparse data - Less interpretable compared to simpler models

Further details on these regression algorithms, including details descriptions of their main features, are provided in the Supporting Information.

## Current status of incorporating DAT for production forecasting

**Fig. 5:**
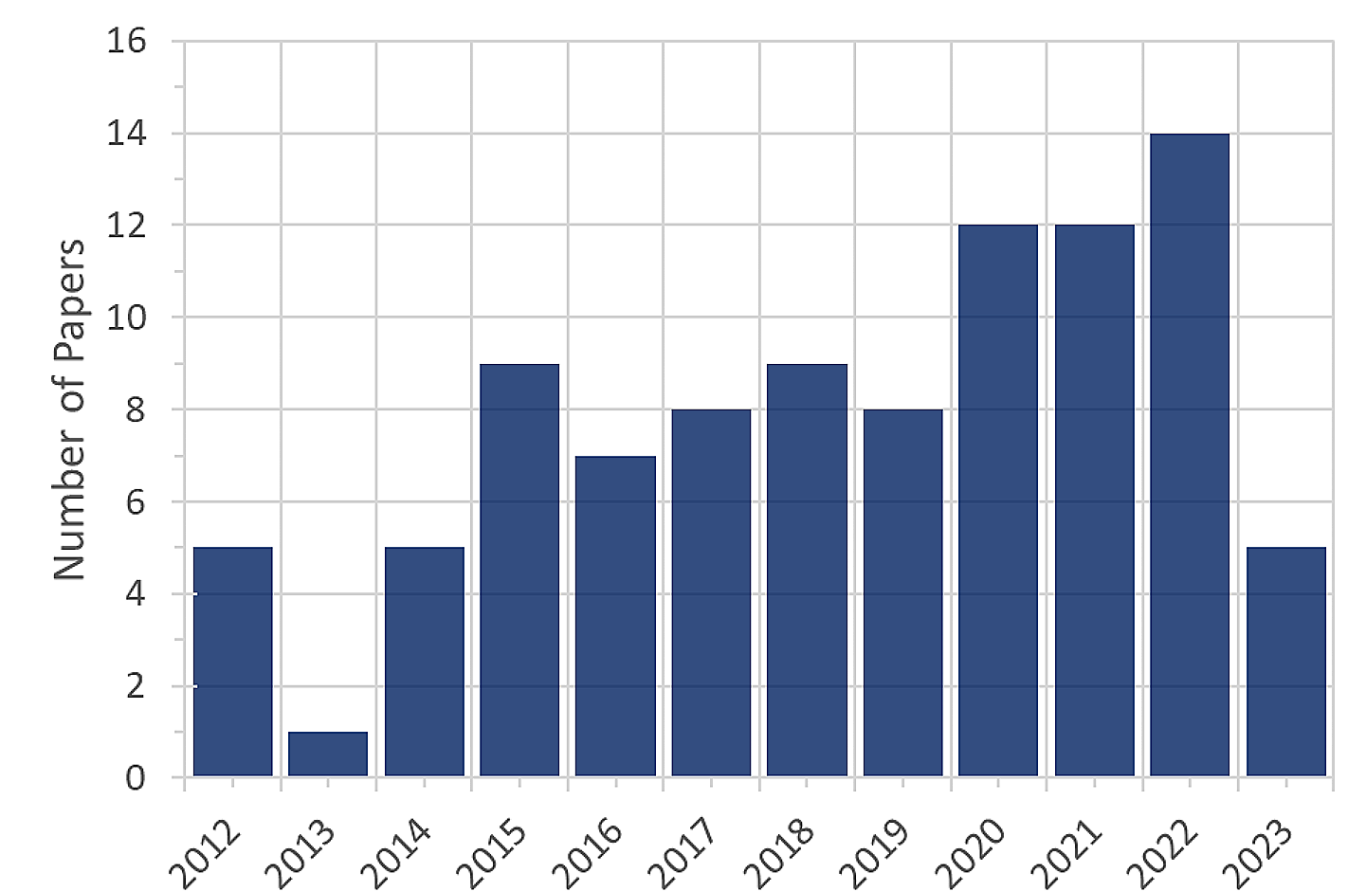
Distribution of the reviewed paper based on the published year

In petroleum engineering, DAT using ML algorithms have been successfully applied to develop models for predicting different rock and fluid properties such as minimum miscibility pressure (MMP), reservoir fluid properties, and relative permeability (Al-Anazi and Gates 2010a, b, c, d, 2012; Alimohammadi et al. 2020; El-Sebakhy et al. 2012; Fathinasab et al. 2015; Ganji-Azad et al. 2014; Olatunji et al. 2011; Rahmanifard et al. 2020, 2021, 2022; Shokrollahi et al. 2013; Tatar et al. 2013). ML algorithms have shown promising performance in predicting reservoir rock and fluid properties, and therefore, many researchers have used these methods to tackle some important problems in unconventional resource management, such as production forecasting and estimated ultimate recovery (EUR). To evaluate the performance of DAT with ML algorithms, we searched google scholar’s website and collected 165 papers that were published in the previous decade and used a dark box approach to forecast production from unconventional reservoirs. After screening out irrelevant papers, we selected 95 research studies, including research and review papers and theses, for a detailed review process. Figure 5 shows the distribution of the reviewed paper according to the published year.

**Fig. 6:**
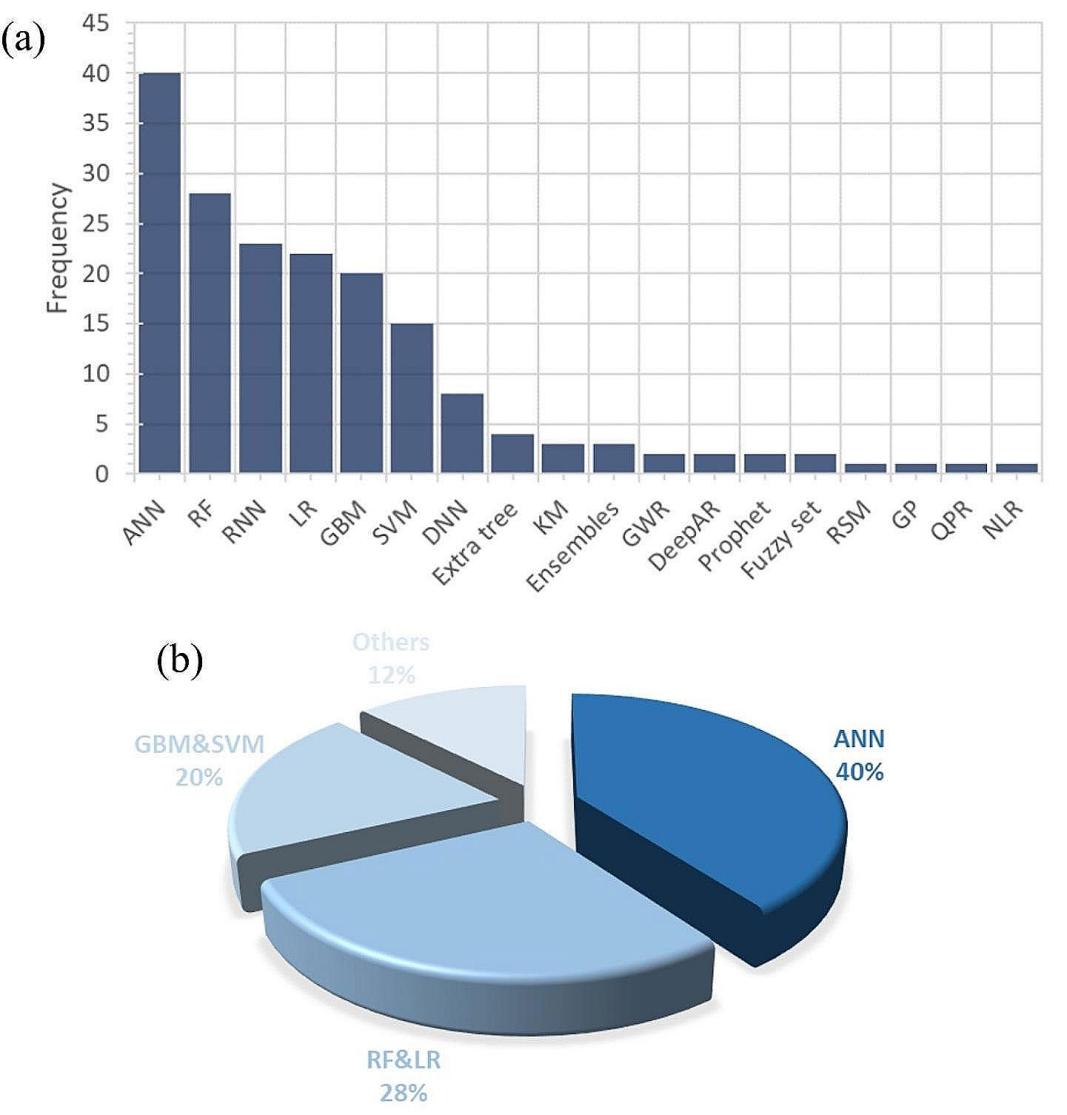
The frequency distribution of the use of ML techniques in the reviewed papers

The frequency and percentage distributions of the ML technique used in the reviewed papers are shown in Fig. 6. Neural network-related techniques^2^ are the most popular methods, followed by LR, and RF methods. Therefore, we group the papers into four broad categories based on the methods they used: NNs, LR and RF, Gradient Boosting Machine (GBM) and Support Vector Machine (SVM), and other methods^3^. For each paper in each category, we provide a brief explanation of the methodology and the results in the following sections. We also discuss more details, such as the detailed description of the algorithms, validation benchmarks, amount of input data, number of wells, formation, and predictive model performance, in the Supporting Information. Note that if a paper compares two or more ML algorithms, we categorize it based on the ML algorithm with the best performance.

### Neural network models

Shelley et al. (2012a) used a feedforward ANN to model the oil recovery and cumulative oil production of 40 horizontal wells in the Bakken Formation (US) based on well and completion design parameters and mud log data. They found that the most important non-controllable parameters affecting well productivity were the contents of butane, total gas, and methane. They also identified the most important controllable parameters as proppant type and amount, treatment fluid type and volume, number of fracture treatments (frac spacing), lateral length and frac staging method.

Shelley et al. (2012b) developed an ANN model using 54 wells in the Eagle Ford formation. The model predicted oil and gas production as a function of well and completion design parameters and mud log data. Their results revealed that increasing the number of frac treatments, reducing treatment volume, and the placement of more proppant with higher conductivity positively affected oil production. In contrast, wells within the gas window responded better to large stimulation volumes.

Using various data sources, Esmaili et al. (2012a, b) created an AI-based model for a Marcellus shale field with 135 wells in different locations. They matched the model’s behaviour to the well location, reservoir properties (e.g., porosity, net thickness, water saturation), completion design parameters (e.g., breakdown pressure, pumped proppant, slurry volume), actual production history (e.g., gas production, flowing head pressure, producing days), and additional parameters (e.g., flow regimes). The production data for the entire field and each well matched the model’s predictions (Esmaili and Mohaghegh 2016).

Used ANN-based models, Enyioha and Ertekin (2014) predicted production rates and bottom-hole pressure profiles (Forward-Acting) and created well designs (Inverse-Acting) for a tight oil formation, depending on well location and well and completion design parameters (e.g., location of the horizontal mainbore, number of laterals, the horizontal mainbore length, direction of each lateral, length of each lateral, the bottomhole producing condition, and spacing between laterals). They used synthetic data to train and test the models. They found that the forward-acting ANN models had low accuracy in forecasting the responses with a wide range in the lower spectrum. They also found that the inverse-acting models had relatively high absolute errors for each predicted well design parameter, even though the predicted well structure matched the desired production profile.

Shelley et al. (2014) combined an ANN model and a genetic algorithm (ANN-GA) to analyze 48 wells in the Marcellus Formation. The model predicted cumulative gas production as a function of reservoir properties and well and completion design parameters. The conducted sensitivity analysis indicated that geology and reservoir quality had a dominant effect on the production profile. Other parameters such as frac volume, proppant amount, fracture spacing, perforation distribution, and fluid volume also affected the well production.

Akbilgic et al. (2015) developed an ANN model for 415 evaluation wells and 365 production/injection wells in Athabasca oil sands reservoirs (Alberta, Canada). The proposed model predicted cumulative steam oil ratio (SOR) of Steam Assisted Gravity Drainage (SAGD) projects as a function of reservoir characteristics, including true vertical depth (TVD), thickness, gamma-ray (GR), porosity, permeability, and oil saturation. The results showed that the developed model predicted the cumulative SOR with high accuracy (R^2^ ≈ 0.8). Also, their sensitivity analysis indicated that cumulative SOR was strongly dependent on the GR, TVD, and permeability.

Amirian et al. (2015) developed ANN-based models using 150 numerical flow simulations. The proposed models predicted the recovery factor (case#1) and Arps’ model parameters (case#2) as a function of reservoir heterogeneities and relevant production/injection parameters, including average porosity, the variance of porosity, the number of shaly layers in each model, average distance of shaly layers to the injection well, the variance of the distance of shaly layers to the injection well, the total thickness of shaly layers, the average thickness of shaly layers, the variance of the thickness of shaly layers, average distance of the shaly layer located at the shortest distance to the injection well, average permeability, the variance of permeability, and shale indicator. Their results showed the acceptable performance of the predictive ANN-based models. They also showed that performing cluster analysis and groupings before ANN modelling enhanced the model prediction capability.

Crnkovic-Friis and Erlandson (2015) presented a deep-learning method using over 800 oil and gas wells in the Eagle Ford Formation. The model predicted EUR and the oil to gas ratio as a function of geological parameters, including porosity, thickness, bulk density, water saturation, total organic carbon (TOC), vitrinite reflectance, and brittleness. They showed that the proposed Deep Neural Network (DNN) model had captured the relationships between geological parameters and the EUR and could be applied to other regions with the same geological principles.

Using mud log data, well and completion design parameters, fluid viscosity, and the top of Formation from the sea level as input variables, Nejad et al. (2015) created a neural network (NN) model that can estimate the cumulative oil and gas production for 81 wells in the Eagle Ford Shale. The model had a high accuracy of over 0.7 R^2^ for the test dataset. The most important factors affecting well productivity were the top of the Eagle Ford Shale from sea level, proppant conductivity, and number of fracture treatments, according to their sensitivity analysis.

Alabboodi and Mohaghegh (2016) used fuzzy pattern recognition, including well quality analysis and fuzzy trend analysis in Marcellus shale as guidelines for developing shale reservoirs and designing hydraulic fracture completions. The input parameters were taken from reservoir characteristics, well and completion data, stimulation data, and geomechanical properties. Their results showed that the fuzzy pattern recognition successfully found the relationships between EUR and reservoir and completion parameters in shale gas reservoirs.

Cao et al. (2016) proposed an ANN model using 2 to 4 years of historical production data in the Eagle Ford formation. Their model predicted hydrocarbon production as a function of geological map data, tubing head pressure (THP), and well locations. They demonstrated that their data-driven approach could complement the empirical or numerical simulation techniques with more confidence.

A deep learning method and Sobol’s sensitivity analysis were used by Wang and Chen (2016) to forecast the 6 months and 18 months cumulative production of 2919 wells (2780 horizontal wells and 139 vertical wells) in the Bakken Formation (Canada). They used various input variables related to well location and geometry, formation thickness, hydraulic fracture characterization, fracturing fluid, and proppant placed per stage. The models had a moderate accuracy of 0.37 to 0.42 R^2^ for the test dataset. The most important factor for the cumulative oil production was the average proppant placed per stage.

Using a minimum of five-year historical production data for three wells, Suhag et al. (2017) provided an ANN model for forecasting oil production in the Bakken Formation (US). The model forecasted oil production as a function of well logs, production, and completion data. Their results showed that their ANN-based model addressed the physical uniqueness of each well with a mean absolute percentage error (MAPE) of 0.95% and a standard deviation of 0.35%.

Using synthetic data (553 and 644 simulations for each model), Enyioha and Ertekin (2017) created two types of ANN models for tight oil reservoirs. The models estimated cumulative oil, gas, water production, and bottom-hole pressure profiles based on various input variables related to rock and fluid properties, reservoir extensions, capillary pressure, relative permeability, and well design and operating conditions. The study demonstrated that the ANN system was a practical, fast, and robust way to predict production performance in tight oil reservoirs.

The work of Li and Han (2017) was an inversion scheme to obtain a set of decline curve parameters for the Logistic Growth Model (LGM) for 100 oil wells using field and synthetic data. The model estimated the DCA parameters as a function of permeability, porosity, fracture width, fracture half-length, fracture conductivity, formation pressure, and formation temperature. The results revealed that the ANN model provided predictions with good accuracy.

Mohaghegh et al. (2017) developed a three-layer, feed-forward neural network to predict the 180 days of cumulative production (Barrel of Oil Equivalent-BOE) for 128 wells in the Marcellus shale Formation. In their model, the target variable is a function of nine input parameters, including TVD, net thickness, porosity, TOC, lateral length, total number of stages, number of clusters per stage, amount of clean volume per foot of lateral length, and the amount of proppant per foot of lateral length. They trained their model with 118 wells and tested it with 10 wells that were not used in the training process. Their model had an average error of 13% for the test wells.

Bowie (2018) applied ML algorithms, such as Multiple Linear Regression (MLR) and ANN, to forecast cumulative gas production for 262 wells in the Duvernay shale Formation. The models forecasted cumulative gas production as a function of completion designs, depths, well spacing, orientations, and liquid yield. They compared the accuracy of the MLR and ANN models for the test dataset and found that the ANN model had a higher variance of 78%, while the MLR model had a lower variance of 67%. They also claimed that their ML algorithms could improve the well performance by 19–97% compared to the existing operator methods.

Luo et al. (2018) suggested an ANN-based model for 2,061 horizontal wells in the Bakken Formation (US). They predicted the first-year production (BOE) as a function of the geologic properties and well-completion strategies. They showed that the normalized volume of proppant, reservoir thickness and depth were the most influential parameters.

Using AI algorithms, such as LR and ANN, Mohammadmoradi et al. (2018) created a data-driven model for an offshore gas condensate platform with 1,600 well-testing data points and 420 days of production history. The model estimated gas and condensate production rates based on wellhead pressure (WHP), choke opening, gas-to-liquid ratio (GLR), and wellhead temperature (WHT). The model performed well in predicting the gas and condensate flow rates for different wells with different potentials and histories.

Sun et al. (2018) implemented a data-driven approach (LSTM model) using the 600 to 800 days historical production rates in the Eagle Ford Formation. The model predicted oil, gas, and water production rates as a function of four-time series, including daily oil, and gas, water production rates and WHP. The results showed that the developed model outperformed the traditional DCA methods with the error varying between 0.1 and 2%.

Using well (e.g., net pay thickness, well location and depth), completion (e.g., injected slick water and proppant volumes, number of clusters, lateral length, wellhead shut-in pressure), and production operation parameters (e.g., the barrel of oil equivalent peak, gas-liquid ratio, initial production rate peak, tubing head pressure, wellhead pressure, initial water production rate, 3-month production rate) as input variables, Han et al. (2019) created an ANN model to estimate the gas production rates for 103 wells in the Eagle Ford Formation. They improved the performance of the ANN model by using the variable importance analysis (VIA), which reduced the number of input variables from 20 to 15. They also clustered the input data into two groups to increase the efficiency of the ANN model. They compared their ANN model with other DCA models (Duong and YM-SEPD) and found that their ANN model had better accuracy for forecasting cumulative production rates.

Using production and shut-in time as two separate time series, Lee et al. (2019) developed an RNN-LSTM model to forecast the 6 and 12-month gas production for 315 gas wells in the Duverney Formation (Canada). They compared their model with using only the production data and the hyperbolic DCA. They found that their model had a higher accuracy when using both time series.

Wang et al. (2019) developed a DNN model for 2,919 wells (2,780 horizontal and 139 vertical wells) in the Bakken Formation (Canada). The developed models forecasted 6-month and 18-month cumulative production as a function of well location and geometry, formation thickness, and well and completion design parameters. The R^2^ of the test dataset for the optimized models varied from 0.71 to 0.72. Their analysis also showed that the average proppant placed per stage was the most influential parameter for 6-month and 18-month cumulative oil production.

Alimohammadi et al. (2020) developed a predictive model using a deep learning approach for the long-term well performance based on 1,085 production data points of a gas well in the Montney Formation. They deployed different RNN models to forecast gas production as a function of several time series, including production rates, tubing-head pressure, and bottom-hole temperature. The results showed that GRU had a better performance compared to other RNNs (LSTM and BRNN). They also showed that RNNs had great predictive capabilities to tolerate the noise and learn multi-domain sequences efficiently.

Chaikine (2020) developed two models, a basic feedforward network (ANN) and a complex convolutional-recurrent hybrid (c-RNN), to forecast the five-year cumulative gas production profiles for 74 horizontal wells in the Montney Formation (Alberta, Canada). He used the mechanical rock properties around perforation clusters, well completion parameters, well spacing, and well completion timing as input variables. He also compared the accuracy of the two models and found that the c-RNN model had a lower MAPE of 14.9%, with the best well having an error of only 1% and the worst well having an error of 83%, while the ANN model had a higher MAPE of 20.6% (Chaikine and Gates 2020, 2021).

Lee (2020) suggested an ANN modelling method using 200 unconventional reservoir simulations for two scenarios: Expanding Solvent SAGD (ES–SAGD) and in-situ upgrading. The model predicted the oil production as a function of fluid cumulative productions during the observation time (the first 540 days with a frequency of 60 days). The model had a reliable prediction performance for the ES–SAGD for bitumen, but a significant discrepancy for the in-situ upgrading. The model improved its prediction performance after using physical insights to select measurable and simple input data.

Song et al. (2020) proposed a hybrid model (LSTM optimized by particle swarm optimization) using synthetic data generated by 351 simulations. The model predicted the daily oil production rate as a function of production data time series. Their results showed that the proposed model outperformed the DCA model, the traditional ANN model, Autoregressive Integrated Moving Average (ARIMA) and Autoregressive Moving Average (ARMA) models.

Zhan et al. (2020) developed an LSTM model to predict the 2-year cumulative oil production for 300 wells in an unconventional onshore asset as a function of the initial 3 months of production data, the tubing pressure, and latency oil rate, The results showed that the developed model was one of the most promising data-driven approaches for the production forecasting challenge with an R^2^ of 0.79 for the whole dataset.

In another study, to predict the first-year average gas production rate in the Montney Formation, Rahmanifard et al. (2020) performed a comparative study among ANN models with different training algorithms to find the best algorithm. They also conducted a sensitivity analysis using the backward regression method to identify the influential parameters. Their results showed that the gas production rate was a strong function of total placed proppant, total fluid pumped, azimuth, total depth, number of fracture stages, completion interval, average treatment pressure, and break-down pressure. Also, a one hidden-layer ANN with the Bayesian regularization training algorithm containing 14 neurons had the best predictions.

Using historical production data as input variables, Fan et al. (2021) applied several data analytics models to forecast oil production rates for three wells in an oil field (China). They created two hybrid models, which combined ARIMA for the linear part and LSTM for the nonlinear part and added a dynamic production (DP) component to account for the nonlinear fluctuations of daily production time profiles due to manual operations. They compared the accuracy of the hybrid models (ARIMA-LSTM-DP and ARIMA-LSTM) with the single methods (ARIMA, LSTM, and LSTM-DP). They found that the hybrid models had more reliable predictive capabilities than the single methods and that the ARIMA-LSTM-DP model performed better than the ARIMA-LSTM model when the production data was influenced by frequent manual operations.

Park et al. (2021) presented a hybrid model combining physics-based reservoir simulations and data-driven approaches using several ML algorithms for more than 20,000 reservoir simulation cases. The model predicted the cumulative oil production as a function of the reservoir, well and completion design parameters (e.g., cluster and well spacing, porosity, water saturation, formation thickness, initial reservoir pressure and temperature, depth, gas oil ratio, oil API, and gas gravity, parameters for relative permeability and dilation/compaction curves). They showed that the optimized ANN model was a good fit for reflecting the complex physics in the reservoir simulations among multiple ML algorithms.

Using a synthetic database of reservoir and geological properties (e.g., porosity, permeability, azimuth, gamma ray, net/gross ration, and caliper) as input variables, Temizel et al. (2021) created a model to better characterize well production profiles with three ML algorithms (SVR, RF, and ANN). They used a grid search strategy to optimize the hyperparameters of ML algorithms. They compared the accuracy of the ML algorithms and found that ANN had a lower mean absolute error (MAE), while SVR and RF had a better performance in datasets with a high standard deviation.

Using randomly generated geological and completion parameters as input variables, Li et al. (2022) created an ML-DCA model with an ANN algorithm to estimate tight oil production performance. They used a numerical model to produce cumulative production profiles. They validated their model with reservoir simulation and found that their model had a good agreement with the simulated production rate and cumulative production. They also analyzed the effect of different parameters on the DCA model and found that fracture spacing and matrix permeability were the most significant, while the monitored oil rate, layer-down and layer-up were the least significant.

Thavarajah et al. (2022) proposed a sequence-to-sequence deep learning-based framework for accurate gas and water production forecasting in the Eagle Ford Formation using three deep learning architectures: mixed input forecaster (MIF), DeepAR, and temporal fusion transformer (TFT). They showed that all three models performed well and captured the general trend of decline. Among the three models, the MIF model obtained the best performance for less production data (short histories), while on histories of longer lengths (more production data), the TFT achieved the best results. They also found that total proppant and total vertical depth were the most influential static parameters of production.

Ning et al. (2022) used three time series forecasting ML methods, including ARIMA, LSTM, and Prophet to predict the oil decline curve in an unconventional shale reservoir. The study used two-year production data from 65 wells located in an unconventional reservoir in the Denver-Julesburg (DJ) Basin. Their results showed that Prophet and LSTM performed better than other algorithms. They also showed that R^2^ was not an appropriate benchmark for evaluating nonlinear regression.

Using supervised (LSTM) and unsupervised (k-mean clustering) ML approaches, Vikara and Khanna (2022) developed a time series predictive model that could forecast natural gas, water, and oil cumulative volumes at the well level. They applied their method to the Spraberry and Wolfcamp Formations. They also used recursive feature elimination with cross validation (RFECV) with an RF algorithm as the estimator to select the final sets of input features for ML models. They found that the production outlook was influenced by well completion, decline curve, and spatial and reservoir attributes.

Chen et al. (2022) proposed an LSTM model for predicting the production time series of two shale gas wells in a Chinese complex shale gas reservoir as a function of historical production. They verified the model accuracy by comparing the model prediction with actual production data and other models (DCA and RTA). Using the proposed model, they also conducted a sensitivity analysis to identify the influences of the shale gas matrix permeability, the reformed volume, and the fracture number parameters on shale gas productivity. Their results indicated that the reformed volume and the fracture parameters had the greatest impact on the productivity of shale gas wells.

Lu et al. (2022) proposed a computational framework using the DNN algorithm for predicting the 1-year and 5-year cumulative shale oil production. The selected input parameters included horizontal well length, stage number, cluster number per stage, fluid volume, proppant volume, and pump rate. The performance of the proposed model was then compared with RF and SVR algorithms. The results revealed that DNN exhibits the best production prediction accuracy. They also coupled DNN with particle swarm optimization (PSO) to determine optimal fracturing parameters of horizontal wells under different reservoir quality. The application of the model to Jimusar shale oil resulted in increased cumulative oil and NPV.

Using four numerical flow simulations of a tight oil SRV to create the first-year daily production time-series database, Rahmanifard et al. (2022) compared the performance of six ML algorithms and two statistical methods. The ML algorithms were multilayer perceptron (MLP), LSTM, Bidirectional LSTM (BiLSTM), convolutional neural network (CNN), Long-term Recurrent Convolutional Network (LRCN), and GRU. The statistical methods were Exponential Smoothing and Seasonal Autoregressive Integrated Moving Average. They found that the statistical methods had higher accuracy than the ML algorithms. They also found that MLP had the lowest mean squared error (MSE) among the ML algorithms.

Qiu et al. (2022) proposed an LSTM model optimized by Bayesian optimization to forecast daily gas production in a tight gas reservoir in the Ordos Basin, China. The proposed model predicts the daily gas production as a function of five features: water production and gas rate, wellhead casing pressure, reservoir temperature, and wellhead tubing pressure. Their results demonstrated that the proposed model could handle sequential data and outperformed ARIMA, ANN, and RNN models.

To compare the effectiveness of different predictive models for shale gas production in the Changning area, China, Li et al. (2023) applied MLR, SVM, RF, and ANN models using geological, engineering, and production data (e.g., porosity, well and fracture lengths, TOC, gas content, stage spacing, number of fracture sections, intensity of fracturing fluid and sand). They discovered that the ANN and RF models were accurate, while the MLR and SVM models had large errors. They recommended the ANN model as the best choice for capturing the complex nonlinear relationships in shale gas production and suggested that fracturing parameters could be adjusted to enhance productivity.

Using ANN models and MINLP, López-Flores et al. (2023) optimized various aspects of shale gas production and water management in the Eagle Ford Formation. They trained the ANN model with six inputs (latitude, longitude, true vertical depth, lateral longitude, total proppant, and total fracture water) and two outputs (cumulative gas production in the first 12 months and flowback water) for each well and used the adaptive hyperband algorithm to fine-tune the ANN model’s hyperparameters. The ANN model achieved high accuracy with MSE less than 0.065 and R^2^ more than 0.92. The MINLP model integrated with the ANN model improved water efficiency and reduced freshwater use for hydraulic fracturing.

Supporting Information gives more details about the papers that used NN models, such as the algorithms, the validation benchmarks, the number of input data, the number of wells, the formation, and the performance of the predictive model.

### LR & RF models

Zhou et al. (2014) conducted an individual variable importance measurement and a multiple regression analysis for 173 wells in the Marcellus Formation, which are classified into three groups, i.e., A1, A2, and B, based on formation thickness and thermal maturity. The model predicted the one-year cumulative gas production as a function of proppant mass, the number of stages, fracture fluid volume, vertical depth, treatment rate, and lateral length. They found that the fracture fluid volume and proppant mass were important parameters for group A1, while for other groups (A2 and B), the number of stages and lateral length were important.

Grujic et al. (2015) developed a predictive model using Kriging and ordinary least squares (OLS) algorithms for 172 wells in one of the most prolific shale plays in North America (US). The predictive model forecasted oil, gas, and water rates as a function of petrophysical and pressure, volume, temperature (PVT) properties, and geographical and completions parameters. They reported a MAPE of 21% for 100 test sets using the proposed functional forecasting framework.

Using multivariate analysis, Williams et al. (2015) presented a data analytics approach for 517 wells in the Three Forks Formation. They evaluated the impacts of geological and completion parameters on the 180-day oil production. Their results indicated that the most significant features were total pounds of pumped proppant, average proppant concentration, number of stages in the lateral, and percent ceramic proppant.

Zhong et al. (2015) implemented several ML methods (i.e., LR, RF, SVM, and GBM) for 476 wells in the Wolfcamp Formation. The models predicted oil production as a function of well and completion designs (e.g., well stages, proppant amount, 12-month cumulative oil production, and peak oil production in 12 months). The results showed that the RF model performed best, which was closely followed by GBM and SVM.

Lolon et al. (2016) compared the ML algorithms, including LR, LR-BIC (Bayesian Information Criterion), LR-AIC (Akaike’s Information Criterion), RF, and GBM for 1,749 wells in Middle Bakken and Three Forks Formations. Their models predicted the cumulative oil production as a function of stage cemented, stage spacing, percentage of ceramic proppant, proppant intensity, frac fluid, water cut, Lower Bakken Shale TOC, and maximum treatment rate. The results revealed that the BIC and AIC models provided the best predictions, while the RF model can be used for variable importance evaluation. Also, they identified the water cut as the most influential predictor of well performance.

Khanal et al. (2017) developed a predictive model using LR with principal component analysis (PCA) for 335 simulations (synthetic data) and 46 wells in the Eagle Ford Formation. The model forecasted gas rate, cumulative gas, and condensate-to-gas ratio (CGR) during the early stages as a function of three principle components from reservoir and completion parameters. They showed that multivariate statistical methods could be used with the existing tools (e.g., reservoir simulation and DCA) for routine and quick analysis. However, they pointed out that understanding the limitations of these methods is essential for the optimal use of these methods.

Using PCA and LR, Zhou (2017) predicted the gas well production with 100 simulations of 2,000 daily historical productions. They showed that the LR model with few principal components accurately captured the unconventional gas well’s decline curve patterns. They also improved the prediction results by adding the k-means clustering. They tested the prediction model and the k-means on 100 gas wells with 45 to 93 monthly historical production in the Eagle Ford Formation and found that the model performed well, and the k-means clustering increased the accuracy.

Using the MLR and RF algorithms, Liang and Zhao (2019) developed three predictive models for 1,069 wells in the Eagle Ford Formation. Their models predicted the oil and gas EURs as a function of petrophysics, completion variables, and spatial information. Their results showed that the two RF models for oil and gas EURs outperformed the MLR model. While the key features were TOC, vitrinite reflectance equivalent (VRE), Poisson ratio, upper eagle ford thickness, well-depth, and lateral length of the horizontal well (CLAT) for MLR model, for RF model, they were production type, well depth, X, Y, CLAT, SPH^4^, thickness (Lower Eagle Ford and Gross), hydrocarbon saturation, VRE, API, pressure gradient, and TOC.

Luo et al. (2019) presented the predictive models using RF and DNN algorithms for 3,600 wells in the Eagle Ford Formation. The models predicted the 6-month cumulative BOE as a function of geological and completion variables. The feature ranking showed that the formation depth, proppant loading, and fluid volume are the most important parameters for predicting the 6-month production. Besides, the RF model outperformed the DNN model with an R^2^ of 62%. The results also revealed that the model performed better in the gas and volatile oil regions than in the black oil region, which was mainly attributed to the hydrocarbon compositions and their characteristics.

Wang and Chen (2019) used four supervised ML algorithms, including NN, SVM, adaptive boosting (AdaBoost), and RF for 3,610 wells in the Montney Formation (Canada). In their work, the 12-month oil production was predicted as a function of well and completion design parameters. Recursive feature elimination identified longitude, latitude, TVD, lateral length, pumped proppant, and injected fluid as the most important features. They also found that the RF model performed the best compared to other methods.

Using RF, GBM, and SVM, Han et al. (2020) predicted gas well productivity in the Eagle Ford Formation from 129 wells. They used well and completion parameters and production data as the input features. They ranked the features by importance and found that initial production rate (3 months), slick water, Peak BOE, TVD, THP, Lateral length, and proppant volume were the most important features. They also found that the RF model was more accurate than the other models and clustering the data into two groups improved the accuracy.

Liao et al. (2020) developed a predictive model using several ML algorithms for 1,286 wells in the Cardium tight oil Formation (Canada). The model forecasted the cumulative oil production at the early stage as a function of geography/petrophysics/engineering parameters. They identified resource density, well location, pumped proppant per length, TVD, total stage count, stimulated length, pumped fluid per length, injection rate, and sand concentration as the sensitive, independent variables. They also showed that the accuracy of the RF model was much higher than other ML algorithms.

Kong et al. (2021) proposed a stacked model using the extreme gradient boosting (XGBoost) algorithm as the base model and LR as the meta-model, which was optimized using the Bayesian optimization algorithm for 519 wells in Duvernay Formation (Canada). The model predicted the first 12-month cumulative production as a function of well and completion fluids, reservoir properties, oil price, and shut-in days. They identified completion length, on-production percentage, fracturing fluid volume/length, condensate/gas ratio, and reservoir pressure as the most important parameters for the first 12-month cumulative production. They also showed that feature importance ranking might change over the production period.

Using RF and Dynamic Production Rescaling (DPR), Li et al. 2020, 2021a) predicted the monthly production performance for over 20,000 wells in the Permian and Appalachian basins (US). The predictor variables include well location, formation, wellbore specifications (e.g., heel, depth), well spacing, completion data (e.g., perforated length, proppant intensity, fracing fluid intensity), and monthly cumulative production. They used DPR to rescale the production data to account for the dynamic changes in well conditions. They showed that their method reduced the error by 15-35% and 30-60% compared to ML-DCA without DPR and the modified Arps DCA, respectively.

Xue et al. (2021) proposed a multi-objective RF (MORF) method using 2,000 simulations. The developed model predicted the dynamic shale gas production rate as a function of hydraulic fracturing and geological properties. Their results showed the developed model’s better predictive capabilities than the multi-output regression chain (MORC). The sensitivity analysis showed that the most influencing parameters were the geological properties, including initial pressure and formation thickness. They also indicated that including the initial peak production rate as one of the input features significantly improved the prediction accuracy.

Bhattacharyya and Vyas (2022) examined the relative influences of several well parameters by developing a model using an RF algorithm to predict the parameters of the Stretched Exponential Decline Model (SEDM) in Bakken Shale oil wells in the US. Their results showed that the most important predictors that affect the decline rate and EUR are initial flow rate, proppant amount, fracturing fluid amount, completion length, tubing pressure, and number of fracturing stages, while the TVD heel-toe difference is the least important parameter.

Jafarov (2022) developed two separate models for predicting DCA parameters and 6-month and 12-month gas cumulative production using MLR and RF algorithms. He used a database consisting of the reservoir and completion parameters, including lateral length, oil, water, and gas saturation, temperature, porosity, gas specific gravity, spacing, proppant amount, number of stages and clusters, and net pay, for 53 shale gas wells in five shale gas fields in the US. Their results showed that for the first model (predicting DCA parameters), the RF model performed better, while for the cumulative gas production prediction, the MLR algorithm outperformed the former one.

Gao et al. (2022) presented a new method for predicting unconventional natural gas well productivity in the Canadian gas field using automated processes as a function of fluid per stage, total fluid, injection rate, formation break pressure, number of stages, pumped proppant, and spacing. They used data preprocessing, sample selection, feature analysis, and model screening to make fast and effective forecasts. They also evaluated different ML methods, such as GB, DT, RF, SVR, and ANN, and compared them with traditional methods. They found that the RF model was the most suitable for the study area.

Johan et al. (2023) proposed a new data-driven approach to optimize completions, which overcame the drawbacks of traditional and costly numerical methods. They trained an RF model to predict the best 3-month (B3) production for ~ 75,000 unconventional wells in Texas using several predictor variables, including depth, lateral length, azimuth, total fluid, fluid intensity, total proppant, proppant intensity, and additional engineering features to account the influence of the neighboring wells and depletion. Following the validation of the ML model’s results, a genetic algorithm was also employed to optimize the completion designs.

Supporting Information contains more information about the papers that used LR and RF methods, such as the algorithms, the validation benchmarks, the number of input data, the number of wells, the formation, and the predictive model performance.

### GB & SVM models

Schuetter et al. (2018, 2015) developed several ML models using LR, RF, GBM. SVM and Kriging Model (KM) algorithms for 476 wells in the Wolfcamp Formation (US). The first 12 months of cumulative oil production were predicted by several input parameters, including well architecture, well locations, and completion design parameters. The results showed that the GBM model had the highest accuracy. They also suggested building a decision tree where multiple input-output models were combined with a statistical averaging approach.

Vyas et al. (2017) developed three predictive models using RF, SVM, and Multivariate Adaptive Regression Splines (MARS) algorithms for several wells in the Eagle Ford Formation. The models predicted the decline curve parameters for several DCA models, including Weibull decline curves, Arps, Duong, and SEDM, as a function of initial flow rate, total proppant amount, total fracturing fluid amount, stages, completed length, TVD of the heel, TVD heel-toe difference, and well location. They found that SEDM with SVM algorithm was the most suitable model. Also, the feature importance ranking showed that initial flow rate, total proppant amount, and TVD as the most influential predictors.

Panja et al. (2018) developed an AI tool with PSO using several ML algorithms, including ANN, Least Square Support Vector Machine (LSSVM), and a second-order polynomial RSM for 144 simulations of a hydraulically fractured low permeability reservoir. The models predicted oil recovery and gas-oil ratio (GOR) as a function of gas relative permeability exponent, matrix permeability, the slope of solution gas-oil ratio versus pressure, initial gas-oil ratio, rock compressibility, initial pressure, fracture spacing, and flowing bottom-hole pressure. They found that LSSVM and RSM had better performance for oil recovery than ANN, while for GOR, LSSVM exhibited the highest accuracy.

Shahkarami et al. (2018) developed several predictive models using four ML algorithms, including SVM, Simple Linear Regression (SLR), Gaussian Process Regressor (GPR), and ANN for 820 wells in the Marcellus Formation. The models predicted the dry gas reservoir performance based on geologic conditions, geographical location, initial production results, and drilling and completion designs. The results revealed that SVM was the most accurate model.

Amr et al. (2018) proposed several data-driven models using seven ML techniques, including ANN, SVM, GBM, LR, RF, and DNN to predict the initial production, EUR, and the initial decline of unconventional horizontal wells for producing and non-producing locations. The predictor variables include well location, reservoir properties (e.g., pay zone thickness), drilling and completion data (e.g., lateral length, proppant amount, water intensity, cumulative oil production), and neighboring well information (cumulation oil production), Their results showed that the proposed ML models outperformed Arps-based estimates.

Using LSTM, XGBoost and DCA, Temizel et al. (2020) estimated EUR in unconventional tight-shale reservoirs for different hydrocarbon types, such as condensate, dry gas, volatile oil, and light oil as a function of historical production rates. They used four reservoir simulation models as the input data. They showed that the input data affected the choice of the best ML algorithm. They also showed that for the input data with a fast decline trend, the DCA methods performed better than the ML methods.

Baki et al. (2021) outlined an overall workflow using three ML algorithms (XGBoost, ANN, and SVR) to explore the primary impact of completion parameters on the production performance of the wells in the Eagle Ford Formation. The input data included well information, location, and completion data (e.g., lateral length, fluid volume, proppant volume). They showed that the XGBoost model was the best among the three algorithms. They also found that the model could measure the influence of the reservoir and geological data on the production performance, especially when the data was limited.

Niu et al. (2022) developed EUR prediction models for shale gas wells using one year of the flowback rate and test production data. They used four ML algorithms, including RF, KNN, SVM, and gradient boosting decision trees and designed four schemes according to the feature importance. Among the four algorithms, the SVM algorithm performed better than the other algorithms. They also found that the predictive model performed better based on the important features compared to all features.

Zhai et al. (2022) collected geological (e.g., permeability, porosity, thickness, pressure, fracture gradient), drilling and completion data (e.g., lateral length, well and stage spacing, proppant amount, slick water, and injection rate) for 384 wells in the Changning field to predict the shale gas well production potential by using four ML methods: XGBoost, RF, ANN, and SVR. They found that ML methods accurately reflected the nonlinear relationship between the influencing factors and production. Also, they showed that the SVR algorithm had smaller errors compared to other algorithms.

Using 1,182 shale core samples from 13 coring wells, Hui et al. (2023) studied the factors that affected the productivity of the Duvernay shale gas reservoir. They used mineralogical, geochemical, petrophysical, and geomechanical variables to characterize the reservoir. They tested four ML methods: RF, ANN, Extra Trees (ET), and Gradient Boosting Decision Tree (GBDT) and found that the ET algorithm was the best with an R^2^ of 0.817 and MSE of 0.225. They also found that the main influential factors were production index, formation pressure, effective porosity, total organic carbon, gas saturation, and shale thickness.

Ren et al. (2023) developed a data-driven framework to forecast future cumulative oil production in unconventional asset development in the Permian basin. They found that Natural Gradient Boosting (NGB), MLP, and RF algorithms gave similar results, which suggested that data quality was more important than ML algorithm choice. They also used the Monte Carlo Chain Regressor to measure uncertainties. They used SHAP (SHapley Additive exPlanations) and LIME (Local Interpretable Model-Agnostic Explanations) to identify the key factors affecting cumulative oil production. The SHAP analysis revealed that the primary factors impacting cumulative oil production are geospatial, operation, completion, well spacing, and another instance of completion. Conversely, the LIME analysis identified drilling, geospatial, start year, and completion as the most significant contributors.

Further details about the papers using GBM and SVM methods, including the algorithms, the validation benchmarks, the number of input data, the number of wells, the formation, and the performance of the predictive model, are provided in the Supporting Information.

### Other models

LaFollette et al. (2012) evaluated feature influence ranking on the gas production rate using boosted tree models for 16,970 wells (12,043 horizontal and 4,368 vertical) in Barnett Shale (US). They found that the most important variables were TVD, Y path, total fracturing fluid volume, average injection rate per stage, and 20/40 mesh proppant. In another study, Gullickson et al. (2014) proposed a tool using a Geographically Weighted Regression (GWR) model for 87 wells in Bakken and Three Forks Formations. The model evaluated the cumulative oil production as a function of completion design parameters (e.g., lateral length, pumped fluid and proppant, completion and proppant types). The results revealed the successful application of the GWR model for explaining the production profiles. They also showed that GWR had better performance than the LR-PCA model. Gupta et al. (2014) described the workflow as a simple tool using data mining and time series analysis for hydrocarbon production forecasting based on historical production data. They showed that the prediction capability of the proposed workflow was strongly dependent on the input data quality.

By applying the Transient Hyperbolic Model (THM) to various flow regimes and creating percentile neighborhood forecasts, type curves, and a revised likelihood algorithm, Fulford et al. (2016, 2015) improved the method of Gong et al. (2011). They tested their ML framework on 136 liquid-rich wells in the Bakken Formations (the Elm Coulee Field) to show its accuracy and calibration. They also used their method to measure the effect of completion design on the rate-time behaviour in the Cleveland sand Formation (235 tight-oil wells) and the Wolfcamp Formation (124 liquid-rich wells). They found that their ML model with well-calibrated bias gave better uncertainty estimation of the forecast’s distribution.

Mohaghegh (2016) presented advanced data-driven analytics to distinguish the impact of well design parameters on gas wells productivity in the Marcellus Formation. He showed thatin low-quality shale, where reservoir properties like gas saturation, net thickness, porosity, and total organic carbon (TOC) are considered, the completion design parameters—such as initial shut-in pressure, number of stages, treatment pressure, pad volume, and proppant—do not carry the same level of significance as they do in high-quality shale. Mohaghegh (2017) also presented a new method (Shale Analytics) for the analysis, modelling, and optimization of shale wells. The proposed approach discovered the patterns in the data and predicted the well productivity as a function of logging, drilling, operational, and completion measurements using ML algorithms. By generating the type-curve, the proposed approach also helped optimize the field development plans and future completions.

To evaluate the influence of the completion design, formation quality, and hydraulic fracturing on the well productivity of a shale asset in Texas, Mohaghegh (2019) presented the Shale Descriptive Supervised Fuzzy Cluster Analysis (SFCA). His results revealed that poor wells had been drilled in poor to average quality parts of the shale formation, while they were completed with small frac jobs, including high volumes of cross-linked gel and high proppant concentration. Primera et al. (2019) discussed a complete overview of the Vaca Muerta and Loma Campana areas (Argentina). They demonstrated the high performance of AI forecasting techniques to speed up the resource estimation processes.

Schuetter et al. (2019) compared the performance of nine ML models using the data from 318 wells in the Wolfcamp Formation (Permian Basin). The models predicted the cumulative oil production within the first year of operation as a function of well and completion parameters, including well location, azimuth, depth, completion year, drift angle, number of stages, and proppant and fluid amounts. The results of the study showed that the NN stacking ensemble model outperformed other models.

Kong et al. (2020) developed a dual-flow-regime decline model using Markov Chain Monte Carlo (MCMC) simulation, based on the Arps DCA model for 344 gas wells in the Montney Formation. The input data consisted of different categories, including drilling, completion parameters, reservoir properties, operational time, and average oil price. Their results revealed the model’s capability to simulate the well performance with a very short production history. Also, the proposed workflow was reliable in identifying single-flow-regime and dual-flow regime profiles and quantifying their decline parameters. Finally, they showed that the proposed method was more accurate compared to bootstrap and deterministic regression methods.

Tadjer et al. (2021) demonstrated DeepAR and Prophet models for 22 wells with 105 to 362 monthly production history in the Midland field (US). The model predicted the short-term oil well performance as a function of historical oil production. They found that DeepAR and Prophet models were reliable procedures for nonlinear short-term forecasting problems. They also suggested the incorporation of physics constraints in the training process of a DNN model to replace or speed up the DCA for the long-term forecast of oil and gas wells.

Hui et al. (2021) developed a comprehensive data analytics approach using several ML algorithms (LR, ANN, ET, GBM) for 573 wells in the Duvernay Formation (Canada). The approach predicted the 12-month gas production as a function of geological and operational parameters, including total proppant mass, fluid injection, permeability, number of stages, well TVD, porosity, y coordinate, formation pressure, gas saturation, x coordinate, horizontal length, formation thickness, and distance to the fault. They also found that the ET model had the highest prediction capacity with an R^2^ of 0.809.

Meng et al. (2022) proposed a hybrid data-driven procedure for dominant factor identification, production forecast, and optimization using the actual production data collected from the Fuling shale gas field (Sichuan Basin, China). The input features include geologic (e.g., depth, TOC, porosity, pressure coefficient, tectonic curvature), drilling (target layer penetration, angle to minimum horizontal stress), and completion (simulated length, number of stages, fluid and proppant intensities, formation break pressure). They integrated the ML methods (RF, GBDT, XGBoost, and ensemble), game theory approach, and optimization algorithms (PSO, Differential Evolution, and Bayesian optimization) in all procedures. Their results showed that geological parameters were the most important factors affecting shale gas production. Besides, the proposed hybrid data-driven procedure provided specific and reliable suggestions for development plan optimization, and it outperformed traditional and experience-based approaches in terms of efficiency and accuracy.

Supporting Information has more information about the papers in this section, such as the algorithms, the validation benchmarks, the number of input data, the number of wells, the formation, and the predictive model performance.

## Discussion

For evaluating the performance of the models developed for forecasting the production in unconventional reservoirs, we created a database from the results of the reviewed papers. The database contains the following parameters: the used ML algorithms, the benchmarks for model evaluation, the formation for which the model was developed, and the type of input data (field data or synthetic data). We only used the benchmarks reported for the test dataset in the database preparation process. If the test dataset was not available, we used the results for the whole dataset.

A significant majority, approximately 81%, of the studies we reviewed relied on field data to train their ML models. This preference is primarily due to the direct correlation between field data and real-world conditions. Field data represents the true behavior of reservoir systems, providing a factual basis for production rates, well performance, and reservoir dynamics. Additionally, the historical perspective offered by field data allows ML models to incorporate past trends into their learning process. However, field data can often be sparse, limited by the number of well locations or the consistency of measurements. The presence of noise can further complicate the accuracy of the models. Additionally, gaps in data collection can disrupt the continuity needed for effective model training. Moreover, privacy and confidentiality agreements impose restrictions on accessing field data (Sheppard 2020).

On the other hand, a smaller fraction of studies, about 19%, used synthetic data generated through reservoir simulations. The controlled environment of simulations allows for the creation of diverse scenarios, which might not be feasible (e.g., experimenting with various well placements and hydraulic fracturing designs). Simulated data is comprehensive, covering a range of variables, and facilitating sensitivity analysis, which are crucial for fine-tuning models. Yet, synthetic data is built on assumptions and simplifications (e.g., homogeneity, linear behavior) that may not fully capture real-world complexities. The accuracy of simulation models is heavily dependent on the quality of input parameters and the underlying physics. Furthermore, simulated results serve as approximations based on model assumptions (Bozzella 2023; Malec 2018).

**Fig. 7:**
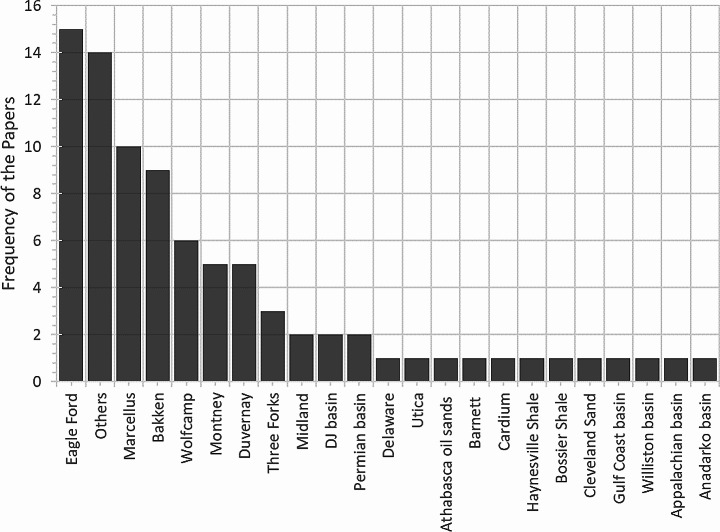
Distribution of the reviewed papers according to the formations those papers where the formation name is out of North America or not clearly stated, are categorized as others

Figure 7 shows the proportion of papers using field data by formation (some papers used developed predictive models for more than one formation). The comparison demonstrates that four formations were the most popular among the papers: Eagle Ford, Marcellus, Bakken, and the Wolfcamp, which may be related to their importance, availability, and variability of the data. These formations represented 18%, 12%, 11%, and 7% of the papers, respectively. The other papers used field data from different formations, such as Three Forks, Duvernay, and Montney.

**Fig. 8:**
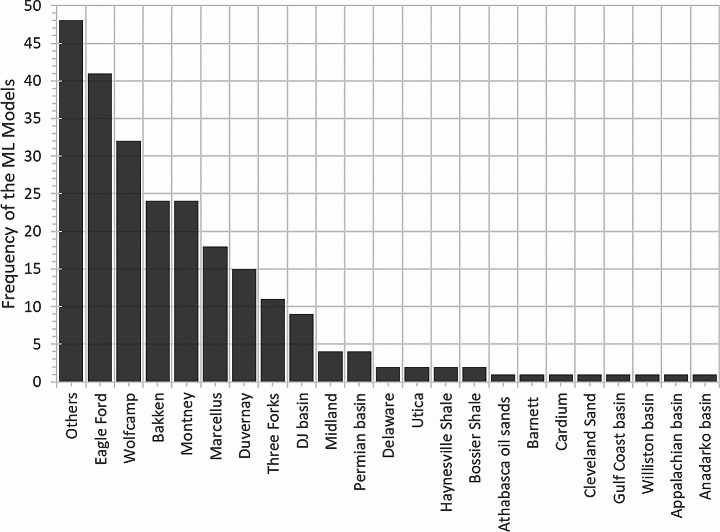
Distribution of developed ML models based on the formations

We found 305 predictive models in the reviewed papers (some papers used more than one ML algorithm to develop predictive models) as shown in Fig. 8. The figure does not include the 59 models that used synthetic data. It reveals that the most common formations for developing predictive models were Eagle Ford, Wolfcamp, Bakken, Montney, Marcellus, and Duvernay, which together accounted for 63% of the models. It also shows that some formations, such as Three Forks, DJ basin, Midland, and Permian, had a relatively low number of models, which may indicate a lack of data or a need for more research.

The reviewed papers used various measures to evaluate the performance of predictive models, such as:

- Mean Absolute Percentage Error (MAPE), which is the average of the absolute percentage errors of the predictions,
- R-squared (R^2^), which is the proportion of the variance in the observed data that is explained by the model,
- Mean Absolute Error (MAE), which is the average of the absolute errors of the predictions,
- Root Mean Squared Error (RMSE), which is the square root of the average of the squared errors of the predictions,
- Mean Squared Error (MSE), which is the average of the squared errors of the predictions,
- Others: some papers used other measures that are less common, such as prediction root mean squared error (PRMSE), symmetric mean absolute percentage error (SMAPE), and mean scaled absolute error (MASE).

**Fig. 9:**
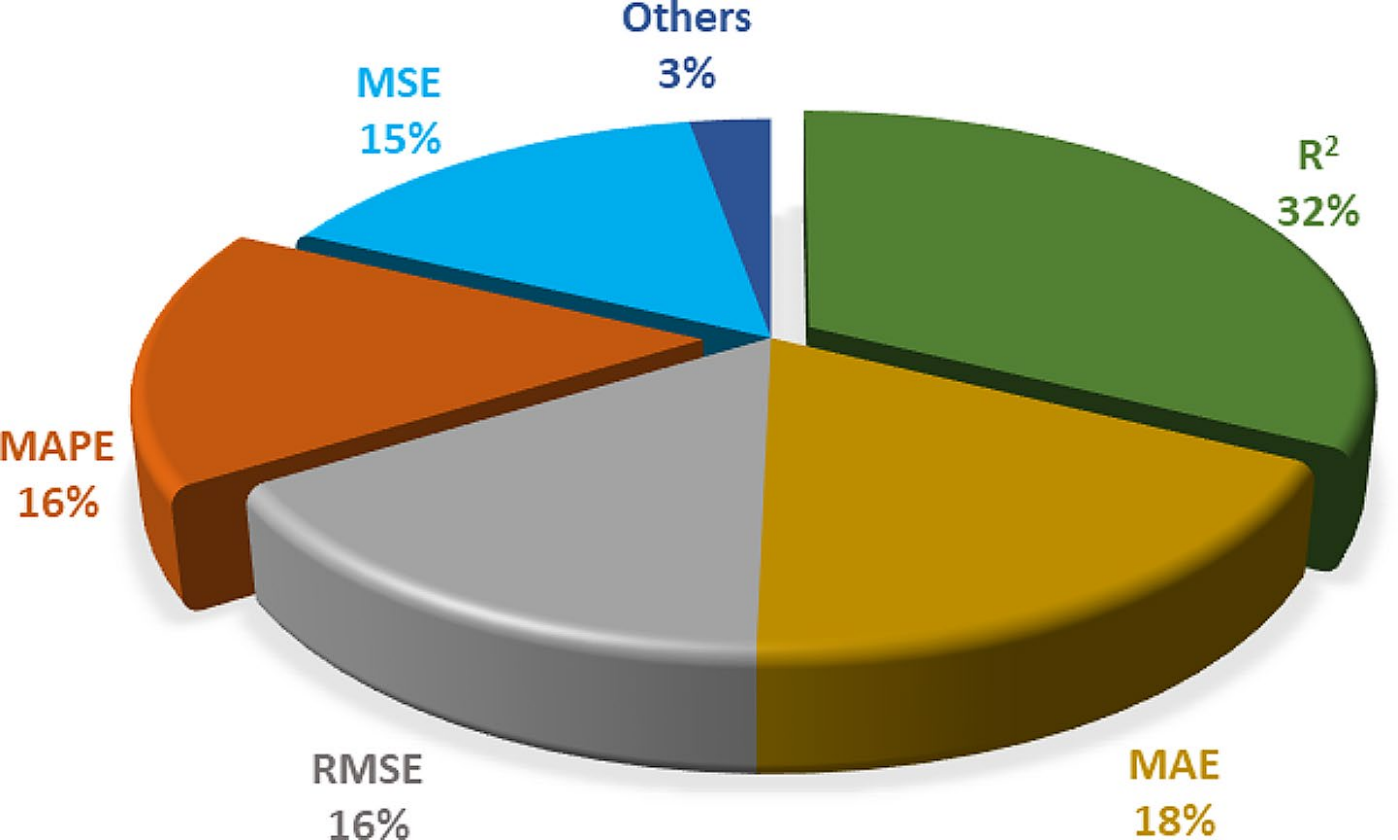
Distribution of the benchmarks for predictive model validation

Most of the reviewed papers used some of these measures to validate the model performance, while a few papers used visualization to show the fit of the model to the data. Figure 9 displays the distribution of the validation measures used in the papers. The figure shows that R^2^ was the most popular measure, followed by MAE, RMSE, and MAPE. The figure also reveals that some measures, such as PRMSE, SMAPE, and MASE, were rarely used, which may indicate a lack of standardization or consensus on the best validation measure for predictive models.

**Fig. 10:**
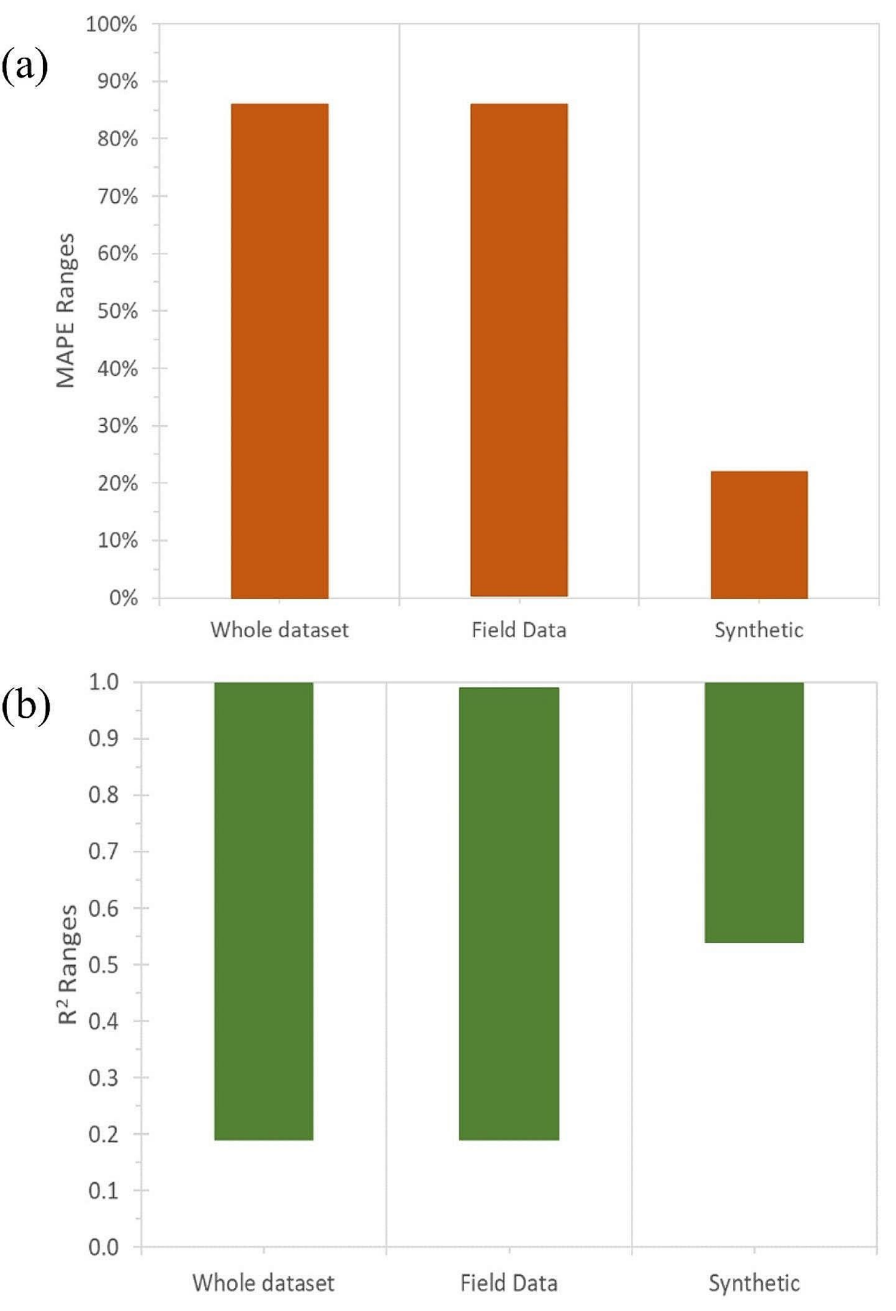
Benchmark ranges for (**a**) MAPE and (**b**) R^2^

We selected R^2^ and MAPE as the benchmarks for evaluating the performance of the predictive models, because they are dimensionlessand comparable across different target functions (oil and gas production rate). Additionally, some papers did not report the units of MAE, RMSE, and MSE, which made them difficult to compare. Figure 10 shows the reported ranges of MAPE and R^2^ for the predictive models developed in the papers. The figure indicates that the MAPE values ranged from 0.4 to 86%, while the R^2^ values ranged from 0.2 to 0.99 for the field data. Moreover, the developed models using the synthetic data show better performance compared to the field data with 0–22% for MAPE and 0.54 to 1 for R^2^.

Predictive models that utilize synthetic data demonstrate superior performance, as evidenced by their lower MAPE and higher R^2^ compared to models using field data. This improvement arises from several key advantages. Synthetic data can be generated with a focus on quality control, ensuring alignment with specific requirements while minimizing common noise and errors inherent in real-world data. Moreover, synthetic data introduces a broader range of scenarios, capturing rare events that historical data might lack (Bozzella 2023).

In contrast, variations in benchmark performance observed in field data may result from differences in data availability and quality. Each reservoir possesses unique characteristics, leading to varying data quality, and representativeness (Sheppard 2020). Models trained on more comprehensive and diverse datasets tend to exhibit better performance. Additionally, the complexity of the geological features and the flow mechanisms in unconventional reservoirs can significantly influence the performance of predictive models (Mohaghegh 2017).

**Fig. 11:**
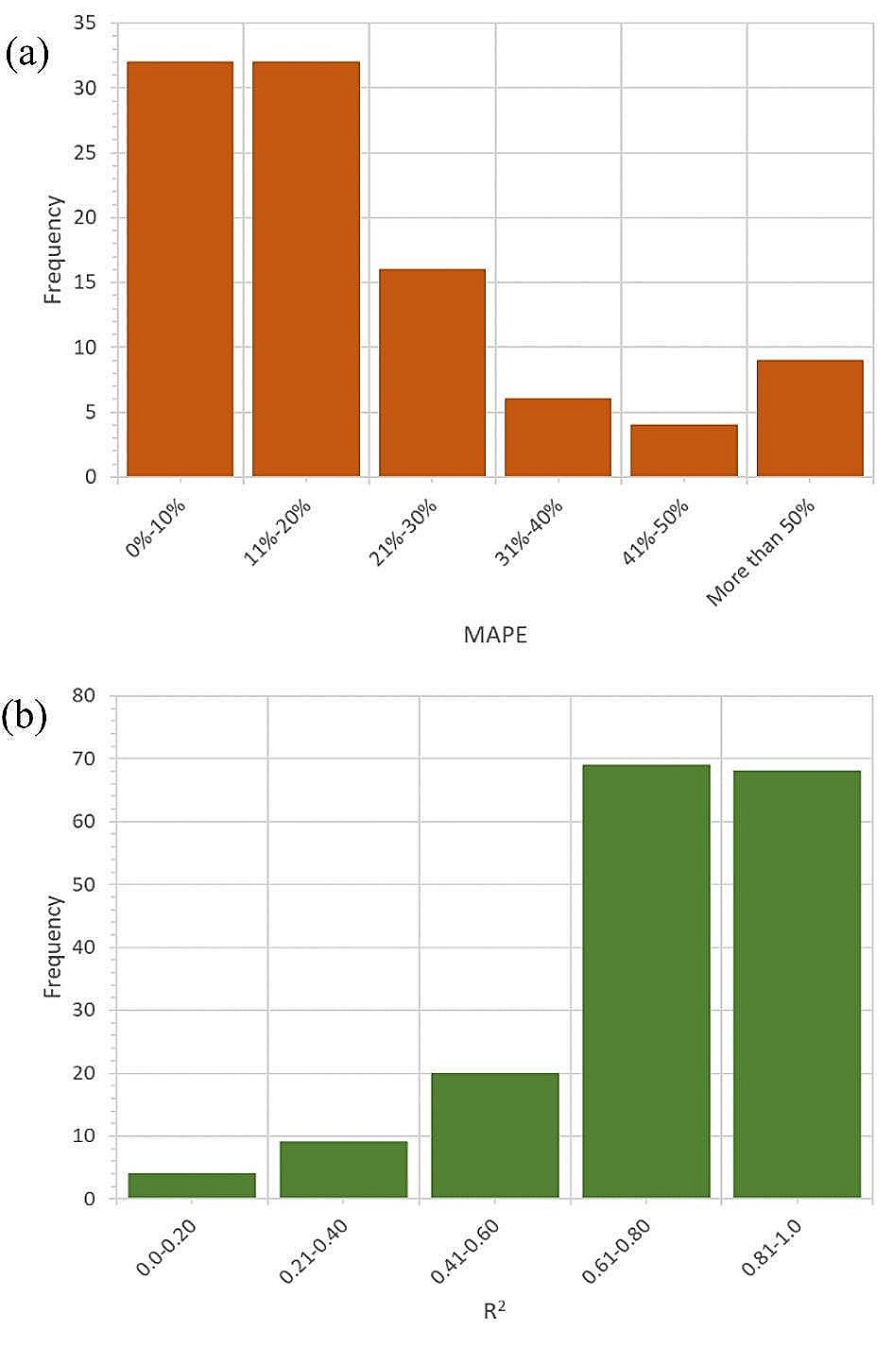
Categorization of the reported results for (**a**) MAPE and (**b**) R^2^

We also categorized the results for each benchmark into different groups to evaluate the performance of the models more easily. We used 6 groups for MAPE and 5 groups for R^2^, as shown in Fig. 11. The figure displays the number of models in each group and the average value of the group. The figure helps us compare how well the models performed using different ML algorithms, input data, and target functions. The results show that the ML models had acceptable performance in terms of MAPE and R^2^, as about 65% of the models had MAPE less than 20% and more than 80% of the models had R^2^ higher than 0.6.

**Fig. 12:**
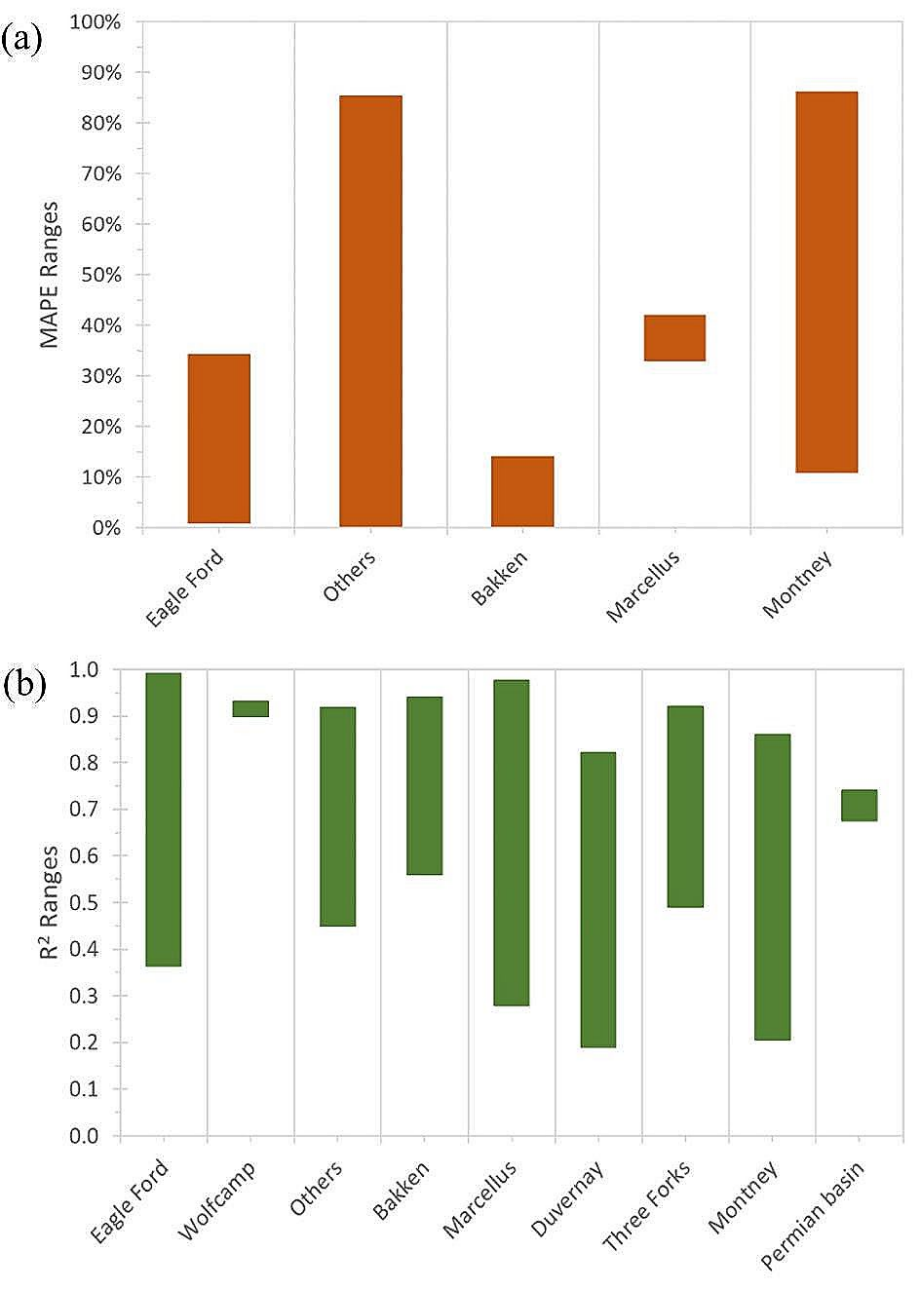
The models’ accuracy and correlation with the field data for (**a**) MAPE and (**b**) R^2^. The data are only for the formations that have more than one record

We also compared the performance of the models that used field data by formation, as shown in Fig. 12. The figure shows the ranges of MAPE and R^2^ values for each formation, which reveals that the models for the Bakken Formation had better performance than the models for other formations in terms of MAPE and R^2^. The models for the Bakken formation had MAPE values between 0.4% and 14% and R^2^ values between 0.56 and 0.94, which were the lowest and the highest among the formations, respectively. It also shows that the models for the Bakken Formation had smaller variation ranges than the models for other formations, which indicates their consistency and reliability (Fig. 12).

## Summary and recommendations

Engineering systems can be classified into two types: clear box systems, which have a sufficient understanding of physics, and dark box systems, which have available data but a poor understanding of system physics. Predicting well production from unconventional reservoirs, which is crucial for developing these assets, is a dark box system. A more practical approach to solving the problem of production forecasting from unconventional reservoirs would rely on data-driven techniques, which can handle the components of the predictive model that are difficult to understand physically. DAT with machine learning (ML) algorithms can learn from data and make predictions without requiring explicit rules or equations.

This paper provided a comprehensive review of the performance of DAT (i.e., ML algorithms) for production forecasting from unconventional reservoirs. We also summarized the main features of the predictive models developed using ML algorithms to forecast production from unconventional reservoirs in several case studies and reports published between 2012 and 2023. We selected 95 research studies, including research and review papers and theses, for the detailed review process. We categorized the selected studies based on the ML algorithm, the formation type, and the performance metrics.

We found that the most used ML algorithms for production forecasting from unconventional reservoirs were NNs, RF & LR, and GBM & SVM, which accounted for 40%, 28%, and 20% of the selected studies, respectively. Other ML algorithms that were used less frequently included DT, DeepAR, and fuzzy logic. We also found that the most common unconventional formation being studied using ML algorithms was the Eagle Ford Formation, which accounted for 18% of the selected studies, followed by Marcellus (12%), Bakken (11%), and Wolfcamp (7%). The results also showed that there was no unique approach for validating the predictive models, and researchers used six different benchmarks, of which R^2^ and MAPE were the most common dimensionless ones. In addition, the ML models had acceptable performance in terms of MAPE and R^2^, as about 65% and 80% of the models had MAPE less than 20% and R^2^ higher than 0.6, respectively. Moreover, we evaluated the models based on the formations and found that the models for the Bakken Formation had better performance.

DAT with ML algorithms have the potential to enhance the reliability and robustness of production forecasting from unconventional reservoirs, as they can learn from the available data and make predictions without requiring detailed knowledge of reservoir physics. However, there are some limitations and caveats that need to be considered when applying them to production forecasting in unconventional reservoirs. These limitations are:

- Data quality and quantity: ML algorithms depend on extensive and dependable datasets for effective training and validation. In the context of unconventional reservoirs, data may often be limited, noisy, inconsistent, or incomplete, potentially compromising the precision and reliability of the predictions. Consequently, rigorous data preprocessing, cleansing, and imputation are vital to improve data integrity and reduce uncertainties.
- Model interpretability and explainability: ML algorithms are often categorized as “black box” models since they do not provide a clear explanation of how they make their predictions or what factors influence their outcomes. This can limit the understanding and confidence of the users and the stakeholders and hinder the practical integration and adoption of these computational methods within the industry. Therefore, it is imperative to employ specialized techniques aimed at providing insights into the logic and the rationale of the ML models, such as the identification of the key features and variables that affect the production performance of unconventional wells.
- Model generalization and transferability: ML algorithms are typically trained and tested on specific datasets, which may not capture the full spectrum of scenarios or conditions found in unconventional reservoirs. This limitation can affect the generalization and transferability of the models, potentially resulting in suboptimal performance when applied to new or unseen data. To address these challenges, there is a need for a comprehensive dataset that accurately reflects the variability and dynamic nature of reservoir and well characteristics over time. Such a dataset will support the model development process, enhancing adaptability and predictive accuracy across a broader range of operational parameters.

Some of the future opportunities in this area include an automated workflow for model development in different reservoirs, new ML algorithm development, a combination of the physics-based model with DAT, and a multi-perspective approach for model comparison.

### Electronic supplementary material

Below is the link to the electronic supplementary material.

Supplementary Material 1

## Data Availability

No datasets were generated or analysed during the current study.

## Nomenclature

AdaBoost: Adaptive boosting
AI: Artificial intelligence
AIC: Akaike’s information criterion
ANN: Artificial neural network
ANN-GA: ANN with genetic algorithm
ARIMA: Autoregressive integrated moving average
ARMA: Autoregressive moving average
BIC: Bayesian information criterion
BiLSTM: Bidirectional long short-term memory
BOE: Barrel of oil equivalent
BRNN: Bidirectional recurrent neural networks
CGR: Condensate-to-gas ratio
CLAT: Lateral length of horizontal well
CNN: Convolutional neural network
c-RNN: Convolutional neural network
DAT: Data analytics techniques
DCA: Decline curve analysis
DJ: Denver-Julesburg
DNN: Deep neural network
DP: Dynamic production
DPR: Dynamic production rescaling
DT: Decision trees
ET: Extra trees
RM ANOVA: Repeated measures analysis of variance
EHF: Explicit hydraulic fracture
ES–SAGD: Expanding solvent steam-assisted gravity drainage
EUR: Estimated ultimate recovery
GBDT: Gradient boosting decision tree
GBM: Gradient boosting machine
GHG: Greenhouse gas
GLR: Gas to liquid ratio
GOR: Gas oil ratio
GPR: Gaussian process regressor
GR: Gamma-ray
GRU: Gated recurrent units
GWR: Geographically weighted regression
KM: Kriging model
LGM: Logistic growth model
LR: Linear regression
LIME: Local interpretable model-agnostic explanations
LRCN: Long-term recurrent convolutional network
LSSVM: Least square support vector machine
LSTM: Long short-term memory
MAE: Mean absolute error
MAPE: Mean absolute percentage error
MARS: Adaptive regression splines
MASE: Mean scaled absolute error
MCMC: Markov-Chain Monte Carlo
MIF: Mixed input forecaster
ML: Machine learning
MLP: Multilayer perceptron
MLR: Multiple linear regression
MMP: Minimum miscibility pressure
MORC: Multi-output regression chain
MORF: Multi-objective random forest
MSE: Mean squared error
NGB: Natural gradient boosting
NLR: Nonlinear regression
NN: Neural network
OLS: Ordinary least squares
PCA: Principle component analysis
PRMSE: Prediction root mean squared error
PSO: Particle swarm optimization
PVT: Pressure, volume, temperature
RF: Random forest
GWR: Geographically weighted regression
RFECV: Recursive feature elimination with cross validation
RMSE: Root mean squared error
RNN: Recurrent neural networks
RSM: Response surface model
RTA: Rate transient analysis
SAGD: Steam-assisted gravity drainage
SEDM: Stretched exponential decline model
SFCA: Supervised fuzzy cluster analysis
SHAP: SHapley Additive exPlanations
SLR: Simple linear regression
SMAPE: Symmetric mean absolute percentage error
SOR: Steam oil ration
SRV: Stimulated reservoir volume
SVM: Support vector machine
SVR: Support vector regression
TFT: Temporal fusion transformer
THM: Transient hyperbolic model
THP: Tubing head pressure
TOC: Total organic carbon
TVD: True vertical depth
VIA: Variable importance analysis
VRE: Vitrinite reflectance equivalent
WHP: Wellhead pressure
WHT: Wellhead temperature
XGB: Extreme gradient boosting
XGBoost: Extreme gradient boosting regressor
YM-SEPD: Modified stretched exponential production decline

## Footnotes

1 There are other techniques for ML algorithms (e.g., reinforcement learning and semi supervised), which are not discussed here.

2 These techniques include conventional Artificial Neural Network (ANN), Deep Neural Network (DNN), and recurrent neural networks (RNN), including Long Short-Term Memory (LSTM), Gated Recurrent Units (GRU), and Bidirectional Recurrent Neural Networks (BRNN).

3 Other methods include Weighted Regression (GWR), Supervised Fuzzy Cluster Analysis (SFCA), Non-Linear Regression (NLR), DeepAR, Response Surface Model (RSM), ensembles, prophet, Kriging Model (KM), Gaussian Process Regression (GPR), Extra Trees (ET), and Decision Trees (DT).

4 SPH is the product of hydrocarbon saturation, porosity, and formation thickness.
