# Supplementary material for: A Comprehensive review of data-driven approaches for forecasting production from unconventional reservoirs: best practices and future directions
Source: Artif Intell Rev. 2024 Jul 22;57(8):213. doi: 10.1007/s10462-024-10865-5 (PMC11263255; doi:10.1007/s10462-024-10865-5)
Supplement: Supplementary file 1 — Supplementary Material 1 [file 10462_2024_10865_MOESM1_ESM.pdf]

A Comprehensive Review of Data-Driven Approaches for Forecasting Production from Unconventional Reservoirs: Best Practices and Future Directions

Hamid Rahmanifard, Ian Gates*

Department of Chemical and Petroleum Engineering, Schulich School of Engineering, University of Calgary, 2500 University Dr. NW, Calgary, T2N 1N4, Alberta, Canada

Supporting Information

S1. Clear Box Methods for Production Forecast in Unconventional Reservoirs

S1-1. Rate Transient Analysis (RTA)

Rate transient analysis (RTA) is a technique that uses the rate and flowing pressure data to estimate and forecast the properties and performance of unconventional reservoirs. It is like pressure transient analysis (PTA), but it also combines type-curve and flow-regime analyses with analytical and numerical simulation (Haskett and Brown, 2005). RTA can help to determine the critical attributes of stimulation and reservoir information, such as hydraulic fracture properties, skin factor, permeability-thickness parameter, and hydrocarbons-in-place (Clarkson, 2013). RTA is an easy-to-use and widely developed approach for unconventional reservoir modelling (Behmanesh et al., 2018; Clarkson, 2013; Ibrahim and Wattenbarger, 2006; Mohaghegh, 2017; Nobakht et al., 2012; Qanbari and Clarkson, 2016). However, it still faces some challenges, such as:

- Dealing with complex fracture geometry (Apaydin et al., 2012),
- Accounting for complex reservoir behaviour, such as non-static permeability and non-Darcy flow effects (Clarkson, 2013; Ozkan et al., 2010),

- Modelling the complex adsorption behaviour of heavier hydrocarbon components for wet gas and gas condensate reservoirs (Qanbari and Clarkson, 2016),
- Adjusting the critical fluid properties in nano-pores (Michel et al., 2012),
- Creating models suitable for multiphase flow modelling and early production forecasts (Clarkson et al., 2012), and
- Identifying the flow regime with noisy data (Clarkson, 2013).

S1-2. Numerical Simulation

Numerical simulation is a technique that uses various models and methods to represent the reservoir and its flow behaviour over time based on physics. It can handle the complex geology and fluid flow dynamics of shale reservoirs. However, it requires a lot of computational resources and time, and the quality of the results depends on the quality of input data and the assumptions made in the model (Mohaghegh, 2017).

S2. Dark Box Methods for Production Forecast in Unconventional Reservoirs

S2-1. Decline Curve Analysis (DCA)

DCA is a method that estimates the future production rates and reserves in unconventional reservoirs by fitting and extending a curve to the historical production data. It assumes that the production decline rate follows a certain pattern, such as exponential, hyperbolic, or harmonic. DCA is a simple and fast method that can give a quick and easy estimate of the production performance and the ultimate recovery of the reservoir. Many methods have been developed for applying DCA to unconventional reservoirs, especially shale gas formations (e.g., Duong,

Extended Exponential Decline Curve Analysis) (Clark, 2011; Duong, 2010; Gupta et al., 2018; Ilk et al., 2008; Valkó, 2009; Valkó and Lee, 2010; Wang et al., 2017; Zhang et al., 2015)

S2-2. Data Analytics Techniques (DAT):

S2-2-1. Neural Networks

A Neural Network (NN) is a computational model that consists of neurons, which are units that perform simple calculations. A Neural Network has an input layer, one or more hidden layers, and an output layer. The neurons in each layer are connected by weights, which determine how much influence one neuron has on another. A Neural Network learns by adjusting the weights based on the data and the desired output (Goyal, 2020; Werbos, 2004). There are different types of Neural Networks, depending on the structure and the function of the layers and the connections. Some of the common types are (Ghoshal, 2020):

- Feed-forward: This is the simplest type of Neural Network, where the information flows only in one direction, from the input layer to the output layer. There are no loops or feedback in the network.
- Multilayer Perceptron (MLP): This is a type of feed-forward Neural Network that has more than one hidden layer. This type of Neural Network can learn more complex and nonlinear patterns in the data, as each hidden layer can extract higher-level features from the previous layer.
- Convolutional: This is a type of Neural Network that has at least one convolutional layer, which is a layer that applies a filter to the input and produces a feature map. The filter can detect the presence and the location of a specific feature in the input, such as an edge or a

shape. This type of Neural Network can reduce the dimensionality and the complexity of the input, as well as capture the spatial relationships among the features. Other main layers are pooling and fully connected.

- Recurrent: This is a type of Neural Network that has at least one recurrent layer, which is a layer that has a memory that stores the information from the previous steps. The recurrent layer can use the memory to influence the current output, as well as update the memory based on the current input. This type of Neural Network can capture the temporal dependencies and the sequential patterns in the data, such as the order or the context.

S2-2-2. Linear Regression

Linear Regression is an ML technique that models a linear relationship between some input and output variables. It tries to find the best values for the coefficients that minimize the sum of the squared errors between the actual and predicted outputs. Depending on the number of input variables, Linear Regression can be classified into two types: simple linear regression (SLR), which has only one input variable, and multiple linear regression (MLR), which has more than one input variable (Brownlee, 2017; Mathworks, 2020).

S2-2-3. Random Forest

Random Forest (RF) is an ML technique that combines the predictions from many decision trees. Each tree in this ensemble is trained on a different subset of the data and uses a different subset of the available features (bootstrap sampling). This way, the model introduces diversity and reduces the risk of overfitting. The final prediction is obtained by averaging the individual predictions made by all trees (Breiman, 2001; Mitchell, 2010; Otero et al., 2017).

S2-2-4. Gradient Boosting Model

Gradient boosting is an ML technique that builds a strong prediction model by combining a series of weak prediction models, usually decision trees. It uses a regularized framework that updates the performance of each weak model by minimizing a loss function (e.g., mean squared error for regression or cross-entropy for classification), which measures the difference between the actual and predicted values of the target variable. Gradient boosting also applies various methods to prevent overfitting, such as shrinking the contribution of each weak model by a factor called the learning rate, limiting the number of weak models or the depth of the decision trees, or adding a penalty term to the loss function (Brownlee, 2020; Chen and Guestrin, 2016; Kaggle, 2020).

S2-2-5. Support Vector Regression

Support Vector Regression (SVR) is a variation of the SVM that is designed for regression problems. SVR's goal is to find a function that fits the data with a small error while keeping the function simple by penalizing large coefficients. SVR uses an epsilon-insensitive loss function, which ignores errors that are within a specified epsilon range. SVR also uses kernel functions to map the data into a higher-dimensional space, where it can be easier to find a linear function. (Dobilas, 2020; Smets et al., 2007; Smola et al., 2004).

S2-2-6. Other ML Models

- Gaussian Process Regression (GPR) / Kriging is a random process that directly infers a distribution over the continuous functions (Ebden, 2008).

- K-Nearest Neighbors (KNN) makes predictions by searching through the entire training set for the K most similar instances and summarizing the output variable for those K instances (the neighbors), which is the mean output variable for regression (Brownlee, 2017).
- Decision Tree is an ML technique that divides the data into smaller groups based on some features, starting from the root node to the leaf nodes, until the target variable is predicted. This technique uses recursive partitioning, which means that each node is a question about a feature and each branch is a possible answer to that question (EDUCBA, 2020).
- Ensembles are ML techniques that combine predictions from multiple models to achieve better accuracy than individual models. There are three main methods to create ensembles: bagging, boosting, and stacking. Bagging trains multiple models on different subsets of the data and averages their predictions. Boosting trains multiple models one after another, where each model tries to correct the errors made by the previous models. Stacking trains multiple models on the same data and uses another model to learn how to best combine their predictions (Brownlee, 2021; Zhou, 2012).
- Extra Trees (Extremely Randomized Trees) are ensemble learning methods that build a set of decision trees from the data. Unlike regular decision trees, which choose the best split at each node, Extra Trees randomly select the split rule at each node. This makes them faster and more robust to noise than regular decision trees. Extra Trees are like Random Forests, but they do not use bootstrap sampling to create the subsets of data for each tree (Geurts et al., 2006).

Detailed descriptions of the abovementioned algorithms are provided in the literature (Alimohammadi et al., 2020; Brownlee, 2018, 2017; Ebden, 2008; Mathworks, 2020; ML Glossary, 2017; Mohr, 2018; Nagpal, 2017; Pedamkar, 2020; Rahmanifard et al., 2020; Rahmanifard and Plaksina, 2019, 2018; scikit-learn, 2020).

S3. Details of Papers with the ANN-Based Predictive Models

References	Objective	Formation	Algorithm & Benchmarks	Specifications	Input data	Database	Results
(Shelley et al., 2012a)	To predict cumulative oil production and oil recovery	US-Bakken	- ANN<br>- R^2	- one hidden layer with 6 neurons for each layer	10 variables	40 horizontal wells (30 for train and 10 for the test)	- R^2 of 0.89 and 0.94 for best month oil cumulative and oil recovery
(Shelley et al., 2012b)	To estimate the first 30-day BOE/psi, BO/psi, and MCF/psi	Eagle Ford	- ANN-GA<br>- R^2	- One hidden layer with 11 neurons for each layer	13 variables	54 horizontal wells (39 for train, 6 for validation and 9 for test)	- R^2 for oil equivalent, oil, and gas are: 0.85, 0.8, 0.87
(Esmaili et al., 2012a, 2012b; Esmaili and Mohaghegh, 2016)	To model and history match the gas production	US-Marcellus Shale Asset in Southwestern Pennsylvania	- ANN<br>- R^2	- One hidden layer with a variable number of neurons	38 variables	135 horizontal wells (80% for train, 10% for calibration, and 10% for verification)	- R^2 of 0.99, 0.97 and 0.975 for training, calibration, and verification<br>- 101 wells with less than 10% error, 22 wells with 10 to 20%, 12 wells with errors higher than 20%.
(Enyioha and Ertekin, 2014)	To forecast oil production rates (Forward-Acting) and generate well designs (Inverse-Acting)	Synthetic Data	- ANN<br>- MAPE	- training algorithm: Scaled Conjugate Gradient<br>- transfer functions: logsig and tansig<br>- 4 layer	7 variables for the production prediction model and Oil cumulative production for well designs	-	Forward-Acting with<br>- Flowrate-Specified Borehole Condition (0.01% - 13.62%)<br>- Pressure-Specified Borehole Condition (0.97%-8.67%)<br><br>Inverse-Acting with

References	Objective	Formation	Algorithm & Benchmarks	Specifications	Input data	Database	Results
							- Flowrate-Specified Borehole Condition (0.0% - 16%)<br>- Pressure-Specified Borehole Condition (2%-22%)
(Shelley et al., 2014)	To predict the best calendar month cumulative gas and the first 30-day cumulative gas	Marcellus Shale Asset in Susquehanna, Pennsylvania	- ANN-GA<br>- R^2	-	10 variables	48 wells (34 wells for train and 14 wells for test)	R^2<br>- the best calendar: 0.87<br>- the first 30-day cumulative gas: 0.82
(Akbilgic et al., 2015)	To predict the cumulative SOR	Athabasca Oil Sands Reservoirs (Alberta)	- ANN<br>- R^2, AE, MAE, MSE, and RPE	- 2 hidden layers with 15 and 10 neurons<br>- activation functions: tansig, logsig, and purelin,	6 parameters	415 evaluation wells and 365 production/injection wells (70% for train, 15% for validation, and 15% for test)	- R^2 for training, validation, and test: 0.799, 0.742, and 0.807<br>- AE, MAE, and MSE are: 0.04, 0.58, and 0.83<br>- RPE of 15.28%
(Amirian et al., 2015)	To forecast the recovery factor (case#1) and Arps model parameters (case#2)	Synthetic Data	- ANN-Cluster with PCA<br>- R^2 and RMSE	- average silhouette value (case#1), K-means and fuzzy c-means (case#2),	Up to 12 variables	150 simulations (75% for train and 25% for test)	Case #1-R^2:<br>- 9 PSs: 0.983-0.998 (train) and 0.78-0.995 (test)<br>- 7 PSs: 0.861-0.998 (train) and 0.745-0.822 (test)<br>- 5 PSs: 0.843-0.998 (train) and 0.812-0.938 (test)

References	Objective	Formation	Algorithm & Benchmarks	Specifications	Input data	Database	Results
							Case#2-RMSE:<br>- 12 PSs: 0.839-0.977 (k means) and 0.974-0.999 (fuzzy)<br>- 6 PSs: 0.845-0.982 (k means) and 0.978-0.998 (fuzzy)
(Crnkovic-Friis and Erlandson, 2015)	To predict EUR and the oil-to-gas ratio	Eagle Ford	- DNN<br>- MAPE	-	7 variables	Over 800 wells, including both oil and dry gas wells	MAPE for EUR: 29.1%, 27.9%, and 33.1% for train, validation, and test
(Nejad et al., 2015)	To evaluate completion and frac strategies for the cumulative oil and gas production per average drawdown	Eagle Ford	- ANN-GA<br>- R^2	-	12 variables	39 for train and validation, and 23 for test	For entire datasets:<br>- BOE/psi with an R^2 of 0.92<br>- BO/psi are highly accurate, with an R^2 of 0.81<br>- BG/psi with an R^2 of 0.87
(Alabboodi and Mohaghegh, 2016)	To find a potential trend between the input parameters and EUR	Marcellus Shale Play	- ANN<br>- momentum, learning rate, and weight decay: 0.8, 0.3, and 0.2	- 1 hidden layer with 28 neurons	34 variables	205 wells (80% for train, 10% for calibration, and 10% for verification)	- Higher TOC, higher EUR<br>- Higher values of Young's Modulus, lower EUR<br>- lower spacing between clusters in horizontal wells, higher EUR<br>- Positive impacts of the thickness and the matrix porosity on EUR,<br>- Reducing the soaking time, better future production of the well

References	Objective	Formation	Algorithm & Benchmarks	Specifications	Input data	Database	Results
(Cao et al., 2016)	To forecast hydrocarbon production	Eagle Ford	- ANN<br>- Visualization	- one hidden layer with 15 neurons for each layer	Three parameters (production history, tubing head pressure, locations)	2-4 years of production history (80% for train and 20% for validation and test)	- Graphical results
(Wang and Chen, 2016)	To predict the 6- and 18-month oil production	Canada-Bakken	- DNN<br>- MAE, MSE, R^2	- 4 to 7 hidden layers with 100 and 200 neurons in each layer<br>- With the advanced initialization approach, dropout technique, batch normalization, Adadelta optimizer, 10-fold cross-validation	12 variables	2919 wells (2780 horizontal wells and 139 Vertical)	For MAE, MSE, and R^2:<br>- 6 months prediction: train: 0.69, 0.80, 0.62 test: 0.90, 1.33, 0.37,<br>- 18 months prediction: train: 1.57, 4.14, 0.59 test: 1.95, 6.16, 0.42.
(Suhag et al., 2017)	To forecast 3 & 6-month oil production rates and cumulative oil production	US-Bakken Shale Play	- ANN<br>- Levenberg Marquardt and Bayesian Regularization as the training algorithms<br>- R^2 and MAPE	- 1 hidden layer with 10 neurons	6 variables	3 wells with a minimum of five-year historical production data	- R^2 is 0.98, 0.97, 0.85, and 0.93 for train, validate, test and the whole dataset.<br>- The error for different production periods (85 to 91 months) varies from -1.5% to -0.56% for cumulative oil production<br>- The error for different production periods (88 to 91 months) varies

References	Objective	Formation	Algorithm & Benchmarks	Specifications	Input data	Database	Results
							from 0.37% to 14.02% for monthly oil production
(Enyioha and Ertekin, 2017)	To predict the performance of advanced well structures in a tight oil reservoir	Synthetic Data	- ANN<br>- MAE	- transfer functions: logsig and tansig<br>- 4 layer	7 variables	553 and 644 data samples for test models 1 and 2	MAE:<br>- Pressure-specified Wellbore: 1.61-1.71<br>- Rate-specified Wellbore: 1.55-2.01
(Li and Han, 2017)	To estimate the parameters of the logistic growth DCA model using the ML technique	Synthetic Data and Field Data	- ANN<br>- R^2 and MSE	- 1 hidden layer with 50 neurons	7 variables	100 oil wells (70% for train, 15% for validation, and 15% for test)	- R^2 for the whole dataset is 0.96<br>- R^2 and MSE 0.917 and 0.013 MSCF/d for field data
(Mohaghegh et al., 2017)	To forecast 180 days cumulative production (BOE)	Marcellus Shale Play	- ANN<br>- R^2	- A three-layer, feed-forward neural network<br>- Backpropagation as the learning algorithm<br>- a momentum of 0.3<br>- a learning rate of 0.1	9 variables	128 wells (100 for train, 28 for test)	R^2 for train and test: 0.96, 0.77
(Bowie, 2018)	To estimate the cumulative	Duvernay	- LR, MLR, ANN<br>- R^2	- One hidden layer with 9 neurons and regularization of 2.5	21 variables	262 horizontal wells (157 for train, 53 for	- R^2 for LR, MLR, and ANN are:<br>Training: 0.41, 0.761, 0.926

References	Objective	Formation	Algorithm & Benchmarks	Specifications	Input data	Database	Results
	gas production					validation, and 52 for test)	Test: 0.19, 0.67, 0.781
(Luo et al., 2018)	To predict the first-year BOE	US - Mid Bakken	- ANN<br>- MSE, MAE, R^2	- 4 hidden layers with 100 neutrons in each layer<br>- Activation function: relu<br>- 5-fold cross-validation	8 variables	2,061 horizontal wells (80% for train and 20% for test)	MSE, MAE, $R^{2:}$<br>- train: 0.004, 0.044, 0.745<br>- test: 0.006, 0.054, 0.614
(Mohammad moradi et al., 2018)	To forecast gas and condensate production rates	An Offshore Gas Condensate Platform	- LR and ANN<br>- MAPE	- 1 hidden layer with 6 neurons	two different sets of input vectors: (1, WHP, S, CGR) and (1, WHP, S, WHT)	1,600 well-testing data points (70% for train, 15% for validation, and 15% for test)	MAPE<br>- LR from 0.89% to 1.89%<br>- ANN from 0.35% to 1.09%
(Sun et al., 2018)	To predict the oil, gas, and water production rates	Eagle Ford	- LSTM<br>- MAPE and RMSE	- 2 hidden layers with 30 and 18 neurons<br>- dropout rate: 0.2<br>- 3 days as previous time steps to reconstruct the inputs	4 time series (daily oil, gas, water production rates and wellhead pressure)	600-800 days history (80% for training, 10% for validating and 10% for the blind test)	The error varies between 0.1% to 2%, with RMSE pf 81.65 and 151.27 for two cases
(Han et al., 2019)	To predict the 30, 36, 42 and 48-month gas production rate	Eagle Ford	- ANN, K-Means<br>- MAE, MSE, MAPE, R^2	- 1 hidden layer with 15 neurons<br>- training algorithm: GDM<br>- The activation function: log-sigmoid linear	20 variables (existing ANN) and 15 variables	- 103 wells (70% for train and 30% for test)<br>- cluster 1: 67 wells (46 for	MAE, MSE, RMSE, MAPE, R^2:<br>- Existing ANN: 2.5, 35.1, 5.7, 17.8%, -<br>- VIA ANN: 1.7, 4.4, 2.1, 15.3%, 0.89<br>- VIA ANN cluster 1:

References	Objective	Formation	Algorithm & Benchmarks	Specifications	Input data	Database	Results
				- the learning rate: 0.02	(VIA ANN)	train and 21 for the test)<br>- cluster 2: 36 wells	0.9, 2.6, 1.6, 7.8%, 0.93<br>- VIA ANN cluster 2: 0.5, 2.0, 1.3, 4.4%, 0.99
(Lee et al., 2019)	To forecast the 6 and 12-month gas production	Duvernay	- RNN-LSTM<br>- RMSE	- the number of neurons: 20,<br>- L2-norm regularization parameter: 0.0001,<br>- the dropout rate: 0.5	2 time-series: production with shut-in time	315 gas wells (300 wells for train and 15 wells for test)	RMSE for test wells:<br>- 6-month: 8.05<br>- 12-month: 11.12
(Wang et al., 2019)	To predict the 6- and 18-month oil production	Canada-Bakken	- DNN with Xavier initialization, dropout technique, batch normalization, Adadelta optimizer, k-fold cross-validation<br>- MAE, MSE, R^2	- 3 hidden layers with 200 neurons in each layer<br>- ReLU as the activation function and the learning rate of 0.005	12 variables	- 2780 horizontal wells (6 months) and 2474 horizontal wells (18 months)<br>- 81% of the data used as the training set and 9% and 10% for validation and test.	For MAE, MSE, and R^2:<br>- 6 months prediction: train: 0.29, 0.14, 0.87 test: 0.46, 0.32, 0.71,<br>- 18 months prediction: train: 0.48, 0.47, 0.94 test: 1.05, 2.00, 0.72.
(Alimohammadi et al., 2020)	To predict the long-term gas well performance	Montney	- LSTM, GRU, BRNN<br>- RMSE, MAE, R^2	- No. of epoch: 4000, 4500, 4500<br>- Batch size: 50, 30, 50<br>- no. of neurons: 120, 100, 120<br>- No. of layers: 3, 3,3	3 variables	1 well (1,085 data points) (80% for train and 20% for test)	RMSE, MAE, R^2:<br>- GRU: 5.1e-4, 0.015, 0.86<br>- LSTM: 1.2e-3, 0.025,0.73<br>-BRNN: 2.3e-3, 0.033, 0.66

References	Objective	Formation	Algorithm & Benchmarks	Specifications	Input data	Database	Results
				- Drop-out: 0.4, 0.3,0.3<br>- Optimizer: adam<br>- Learning rate: 0.001<br>- Activation function: relu			
(Chaikine, 2020)	predictions of the five-year cumulative gas production profiles in multistage hydraulically fractured wells	Montney (Alberta)	- ANN, C-RNN<br>- MAPE, MAE	- C-RNN: Input (1), Conv×2 (64), Max Pooling (-), Merge (32), Conv (50), GRU×2 (50) with recurrent dropout of 0.5<br>- ANN: two hidden layer network	10 variables	74 horizontal wells (58 for train and 16 for the test)	MAPE of 14.9%, with the best well for C-RNN, compared to the ANN with MAPE of 20.6%
(Lee, 2020)	To predict the cumulative production for ES–SAGD and the in-situ upgrading of oil shale	Synthetic Data	- ANN<br>- MAPE	- 1 hidden layer	Cumulative production from t = 0 to 540 days, for ES-SAGD and from t = 0 to 120 for in-situ upgrading	200 simulations with 1080 days for ES-SAGD and 250 days for in-situ upgrading (70% for train, 15% for validation, and 15% for test)	- MAPE for ES-SAGD from 0.14% to 0.89%<br>- MAPE for in-situ upgrading from 0.002% to 1.68%
(Song et al., 2020)	To predict the daily oil production rate	Synthetic Data	- RNN, LSTM-PSO, ANN<br>- MAPE, MAE, RMSE	- 3 hidden layers with 11 neurons each	Production data time series	351 samples (80% for train and 20% for test)	MAPE, MAE, RMSE<br>- ANN: 20.14%, 3.46, 4.10<br>- RNN: 18.49%, 3.18, 3.81

References	Objective	Formation	Algorithm & Benchmarks	Specifications	Input data	Database	Results
							- LSTM: 9.52%, 1.37, 1.74
(Zhan et al., 2020)	To predict the 2-year cumulative oil production	An Unconventional Asset	- LSTM<br>- R^2	- 3 recurrent layer	2 time series (production rates and wellhead pressure)	300 wells with<br>- training: the first 3 months<br>- test: the next 21 months	R^2<br>- LSTM: 0.79<br>- Hyperbolic DCA: 0.22
(Rahmanifard et al., 2020)	To predict the first-year average gas production rate	Montney	- ANN<br>- R^2, MAE, MRE, RMSE	-	8 features	603 horizontal wells<br>- 85% for training and validation and 15% for test	a one-hidden-layer ANN with the BR training algorithm and tan-sigmoid and linear activation functions containing 14 neurons in the hidden layer provided the best predictions
(Fan et al., 2021)	To predict the oil production rate	An Oilfield in China	- LSTM, LSTM-DP, ARIMA-LSTM, ARIMA-LSTM-DP<br>- MAPE, MAE, RMSE	- mini-batch size: 20,<br>- optimization function: Adam,<br>- dropout rate: 0.2,<br>- L2-norm regularization parameter: 0.0001,<br>- initial learning rate: 0.005	- Well 1: 2476 samples<br>- Well 2: 1608 samples<br>- Well 3: 687 samples	3 wells (80% for train and 20% for test)	The compound model of ARIMA-LSTM-DP exhibits the lowest RMSE, MAPE, and MAE
(Park et al., 2021)	To predict cumulative oil production	Synthetic Data	- LR, GBM, ANN, RF<br>- R^2	- 2nd-degree poly nominal for LR<br>- 3 hidden layers with 90 neurons	26 variables	20,000 simulations (75% for train and 25% for validation)	R^2 for training and validation dataset<br>- LR: 91%, 90%<br>- GBM: 89%, 85%<br>- ANN: 96%, 94%<br>- RF: 96%, 81%

References	Objective	Formation	Algorithm & Benchmarks	Specifications	Input data	Database	Results
				- 100 boosting stages and 10 trees			
(Temizel et al., 2021)	to predict the cumulative oil performance	Synthetic Data	- SVR, RF, ANN<br>- MAE<br>- Grid search strategy for hyperparameter tuning	- ANN: 2 hidden layers with 8 and 3 neurons and the activation functions: linear, relu, relu, linear<br>- RF: decision trees (50 to 100,000)<br>- SVR: kernel (radial, polynomial, sigmoid, linear), regularization (0.001-100), and epsilon (0.001-1)	8 independent variables	- 100 simulations<br>- 80% for training and 20% for test	MAE:<br>- SVR: 0.07-0.011<br>- RF: 0.11 to 0.13<br>- ANN: 0.059
(Li et al., 2022)	Predict the parameters for Arps decline curve mode for tight oil wells	Synthetic Data	- 3 ANN with Bayesian regularization<br>- R^2	- Hidden layer neurons: 40, 50, 60 for qi, b, Di	- 15 geological and completion parameters	- 10,000 groups of reservoir/ completion<br>- 70% for training, 30% for testing	R^2 of the three Arps decline curve factors (qi, b, Di): 0.966, 0.990, and 0.945
(Thavarajah et al., 2022)	To forecast monthly gas and water rates	Eagle Ford	- MIF, DeepAR, and TFT<br>- R^2, MAE, MASE	- 4-fold cross-validation<br>- 192 unique configurations	For 208 dry gas wells three types of input features:<br>-static features, time-	166 wells for training and 42 wells for test	The average performance of TFT, MIF, and DeepAR for 10-step forecasts for gas and water rates and different historical lengths (1 to 30):

References	Objective	Formation	Algorithm & Benchmarks	Specifications	Input data	Database	Results
					variant variables, and historical production		- MAE: 372.2 363.1 564.9<br>MASE: 0.582 0.674 0.854<br>R^2: 0.536 0.536 0.364
(Ning et al., 2022)	predict a typical well's oil decline curve	The Denver-Julesburg (DJ) Basin	- ARIMA, LSTM, Prophet, Arps, and Reservoir Simulation<br>- For LSTM: 70% for training and 30% for test<br>- RMSE, MAE	- LSTM: 1 input layer, 1 hidden with 4 memory cells and 1 dense layer<br>- Prophet1&2: changepoint_prior_scale = 1 seasonality_prior_scale = 0.001 & 0.002<br>- ARIMA: p, d, q of 0,1,1<br>- Arps: qi, Di and b is 453, 0.014, 0.9	65 oil wells	2-year production forecast	For a typical well ARIMA, LSTM, Prophet1 &2, Arps, and Reservoir simulation:<br><br>- RMSE: 4.97, 2.96, 2.32, 2.63, 3.24, 4.03<br>- MAE: 4.37, 2.19, 1.34, 1.75, 2.47, 3.26
(Vikara and Khanna, 2022)	To predict produced water and natural gas volumes associated with oil production	Spraberry and Wolfcamp	- RFECV with 5-fold and RF algorithm, k-means clustering, LSTM<br>- MSE, RMSE, R^2	-LSTM consists of 4 hidden layers 2 stacked LSTM 2 dense layers with Adam algorithm & sigmoid activation<br>- Learning rate of 0.0001	12 features	5,561 wells (80%, 10%, and 10% for training, validation, and testing)	Monthly gas and water production and joint prediction:<br><br>Training Dataset:<br>- R^2: 0.930, 0.914, 0.922<br>- MSE: 7.63×10^6, 6.72×10^6, 7.17×10^6<br>- RMSE: 2762, 2593, 2679<br><br>Test Dataset:

References	Objective	Formation	Algorithm & Benchmarks	Specifications	Input data	Database	Results
							- R^2: 0.931, 0.899, 0.915<br>- MSE: 7.54×10^6, 7.35×10^6, 7.44×10^6<br>- RMSE: 2746, 2710, 2728
(Chen et al., 2022)	To predict the shale gas production	China	- LSTM<br>- MSE, RMSE, MAE, MAPE	- number of hidden layers: 200<br>- neurons: 2<br>- batch size: 32<br>- learning rate: 0.005	2 wells production data	712 daily production data for each well, including 572 training datasets and 140 validation datasets,	MSE, RMSE, MAE, MAPE for:<br>- well#1: 49388.42, 222.235, 214.4188, 2.332%,<br>- well#2: 131924.5, 363.2141, 249.4259, 4.472%
(Lu et al., 2022)	To predict 1-year and 5-year cumulative shale oil production	Synthetic Data	- DNN, RF, SVR<br>- MAE, MSE, R^2	- DNN: learning rate: 0.003, ReLU, No. of hidden layers: 3, neurons for each layer: 150<br>- 10-fold cross-validation	8 parameters	841 numerical simulations: 80% for training and 20% for test	Training, validation, Testing:<br><br>DNN-One year:<br>MAE: 0.26, 0.35, 0.83<br>MSE: 0.14, 0.21, 0.65<br>R^2: 0.90, 0.85, 0.80<br><br>DNN-Five year:<br>MAE: 0.48, 0.63, 0.74<br>MSE: 0.33, 0.41, 0.45<br>R^2: 0.92, 0.87, 0.84<br><br>RF-One year:<br>MAE: 0.68, 0.94, 1.17<br>MSE: 0.54, 0.82, 1.95<br>R^2: 0.87, 0.83, 0.75<br><br>SVR-One year:<br>MAE: 0.69, 0.87, 1.68

References	Objective	Formation	Algorithm & Benchmarks	Specifications	Input data	Database	Results
							MSE: 0.57, 0.81, 2.31<br>R^2: 0.85, 0.80, 0.72
(Rahmanifard et al., 2022)	To predict the oil production profiles	Synthetic Data	- MLP, LSTM, BiLSTM, CNN, LRCN, GRU, ETS, SARIMA<br>- MSE, time	-	4 production profiles	1-year daily production for four SRVs<br>- 80% for training and 20% for test	the traditional statistical methods have the dual advantages of computational simplicity and effectiveness compared to ML methods
(Qiu et al., 2022)	To forecast the daily gas production rate	Ordos Basin, China	- ARIMA, ANN, RNN, and LSTM<br>- MSE	- LSTM layer: 1<br>- No. of neurons: 20<br>- Learning rate: 0.0001<br>- Dropout: 0.5<br>Number of epochs: 100	5 features	- 81 gas wells (75 for train and 6 for test)	For ARIMA, ANN, RNN, and LSTM:<br>- MSE: 0.29, 0.21, 0.09, 0.006
(Li et al., 2023)	Gas production	Changning Gas Block in Southwest China	- MLR, SVM, RF, ANN<br>- MAPE	-	7 features	- 85 wells<br>- training set (80%) and testing set (20%)	MAPE for training & testing:<br>- MLR: 24.27%, 15.17%<br>- SVM: 16.06%, 24.28%<br>- RF: 12.87%, 12.43%<br>- ANN: 7.9%, 4.53%
(López-Flores et al., 2023)	First 12-months cumulative gas production and flowback water	Eagle Ford	- ANN<br>- MSE, MAE, MAPE, R^2	- Three hidden layers<br>- The no. of neurons in the first, second, and third hidden layers is 30, 100, and 50<br>- The learning rate is 0.0073	- 6 features	- 298 horizontal shale gas well<br>- 85% for the training set and 15% for the validation set.	MSE, MAE, MAPE, R^2:<br>- Total cumulative gas: training (0.06654, 0.14856, 9.68911, 0.93455), validation (0.06444, 0.15447, 9.14203, 0.92664)

References	Objective	Formation	Algorithm & Benchmarks	Specifications	Input data	Database	Results
				- ReLU, Adam			- Flowback water: training (0.04524, 0.13764, 31.4381, 0.95455), validation (0.05463, 0.16458, 34.0849, 0.94548)

S4. Details of Papers with the LR&RF-Based Predictive Models

Authors	Objective	Formation	Algorithm & Benchmarks	Specifications	Input data	Database	Results
(Zhou et al., 2014)	To predict one-year cumulative gas production	Marcellus Shale Play	- LR with PCA and K-means	-	6 variables	173 horizontal wells	Based on the multiple regression analysis and individual variable importance measurement in each geological group, the number of hydraulic fracture stages is found to be the most significant parameter among all factors studied
(Grujic et al., 2015)	To forecast oil, gas, and water rates	US- One of the most prolific shale plays in North America	- Kriging and OLS<br>- MAPE	- Generalized cross-validation using 10 B-spline basis functions with the smoothing penalty on the second derivative (λ = 15)	18 variables	172 horizontal wells (80% for train and 20% for test)	- MAPE of 21% and 22% for OLS and Kriging<br>- data within 95% confidence band 86% and 47% for OLS and Kriging
(Williams et al., 2015)	To evaluate the impacts of geological and	Three Forks	- non-linear multiple regression	-	10 variables	517 wells	R^2: 0.728

Authors	Objective	Formation	Algorithm & Benchmarks	Specifications	Input data	Database	Results
	completion variables on the 180-day oil production		- R^2				
(Zhong et al., 2015)	To predict the cumulative oil production, the maximum monthly oil production, and derived production efficiency	Wolfcamp (Permian Basin)	- LR, RF, SVM, and GBM<br>- MAE and MSE	-	12 variables	476 horizontal wells	MAE (k bbl) and MSE (k bbl^2) for 1st 12 months cum oil:<br>- LR: 30.52, 1743<br>- SVM: 19.14, 1017.95<br>- RF: 11.25, 248.32<br>- GBM: 13.95, 333.68<br>MAE (k bbl) and MSE (k bbl^2) for the best month in the 1st 12 months:<br>- LR: 5.72, 61.52<br>- SVM: 3.69, 37.77<br>- RF: 2.09, 9.21<br>- GBM: 3.8, 25.59<br>MAE (bbl/ft) and MSE $(bbl/ft)^2$:<br>- LR: 1.3, 3.05<br>- SVM: 0.83, 1.89

Authors	Objective	Formation	Algorithm & Benchmarks	Specifications	Input data	Database	Results
							- RF: 0.46, 0.42<br>- GBM: 0.89, 1.38
(Lolon et al., 2016)	To predict 180-day cumulative oil production /ft	Middle Bakken and Three Forks	- LR, RF, GBM<br>- R^2 and RMSE	-	8 variables	1,305 horizontal wells for middle Bakken and 444 wells for three forks	R^2 and RMSE (BO/ft) for train and test:<br>Middle Bakken<br>- LR1: 0.57, 1.632, 1.641<br>- LR2: 0.63, 1.521, 1.595<br>- LR_BIC: 0.62, 1.545, 1.570<br>- LR_AIC: 0.63, 1.527, 1.565<br>- RF: 0.63, 1.538, 1.544<br>- GBM: 0.89, 0.836, 1.543<br>Three Forks<br>- LR1: 0.54, 1.745, 1.897<br>- LR2: 0.7, 1.408, 1.802<br>- LR_BIC: 0.67, 1.438, 1.692<br>- LR_AIC: 0.69, 1.421, 1.688<br>- RF: 0.49, 1.840, 1.959<br>- GBM: 0.92, 0.757, 1.910
(Khanal et al., 2017)	To forecast the gas rate,	Synthetic Data and Eagle Ford	- LR with PCA<br>- MAPE	-	7 variables	335 simulations 46 wells	- simulated data: four PCs

Authors	Objective	Formation	Algorithm & Benchmarks	Specifications	Input data	Database	Results
	cumulative gas, and condensate-to-gas ratio						were enough to yield the prediction with an average error of 0.16%, 0% and 0.77%, respectively, for gas rate, cumulative gas and CGR.<br>- field data: three PCs yielded the best prediction with an average error of 1.63% and 2.98% for gas rate and oil rate, respectively
(Zhou, 2017)	Estimation of the unconventional gas well production	Synthetic Data and Eagle Ford	- PCA, LR, and k-means clustering<br>- R^2 and visual comparison	-	Daily production	- 100 simulations, with 2000 days of production history for the simulation database<br>- 100 gas wells with 45 to 93 months of production history (Eagle Ford)	overall prediction result is satisfying for the real field data, while for synthetic database, average prediction results with an R^2 of 0.97 is reported, which enhances using k-means clustering

Authors	Objective	Formation	Algorithm & Benchmarks	Specifications	Input data	Database	Results
(Liang and Zhao, 2019)	To predict the oil and gas EUR for shale formation	Eagle Ford	- RF and MLR<br>- R^2	- RF for overall EUR with ntree: 500 and sample size: 8<br>- RF for oil EUR: ntree: 500 Mtry: 15 sampsize: 0.8 nodesize: 5<br>- RF for gas EUR: ntree: 500 Mtry: 5 sampsize: 0.8 nodesize: 3	25 variables	1,069 wells (802 for train and 567 for test)	- Overall EUR R^2 for RF is 57%, while for multivariate regression is 49%<br>- R^2 for RF is 60% in the Oil EUR model and 76% in the Gas EUR model
(Luo et al., 2019)	To forecast the first 6-month cumulative BOE	Eagle Ford	- RF, DNN<br>- R^2 and RMSE	- decision trees: 1000	12 variables	3,600 wells (80% for train and 20% for test)	R^2 and RMSE (bbl):<br>RF<br>- train: 0.64, 36,145<br>- test: 0.62, 42,497<br>DNN<br>- train: 0.61, 39,829<br>- test: 0.54, 46,672
(Wang and Chen, 2019)	To predict 12-month BOE	Montney	- RF, AdaBoost, SVM, and ANN<br>- MSE, R^2	- 500 number of decision trees for RF and AdaBoost,<br>- SVM with radial basis function and kernel	11 variables	3610 horizontal wells (90% for train, 10% for test)	for RF, AdaBoost, SVM, NN:<br>- MSEs are: 0.0532, 0.0002, 0.4375, 0.4242 (training), 0.3659, 0.3851,

Authors	Objective	Formation	Algorithm & Benchmarks	Specifications	Input data	Database	Results
				parameter of 0.1,<br>- ANN with three hidden layers, 200 neurons, and ReLU as the activation function<br>- 9-fold cross-validation			0.6260, 0.4910 (test)<br>- R^2 are: 0.9468, 0.9998, 0.5621, 0.5758 (training), 0.6310, 0.6131, 0.3736, 0.5064 (test)
(Han et al., 2020)	To predict the productivity of shale gas reservoirs	Eagle Ford	- RF, GBM, SVM, and K-means for clustering<br>- MAE, MSE, RMSE, MAPE, R^2	- RF: mtry: 5, ntree: 300<br>- GBM: mtry: 5, ntree: 20<br>- SVM: Penalty function: 600, Kernel (RBF): 20<br>- input features ranking based on VIM %IncMSE or IncNodePurity	9 variables	129 wells (80% for train and 20% for test)	RMSE, MAPE, R^2 of trained model<br>- RF (before VIM), 0.639, 22.94%, 0.4<br>-RF (VIM %IncMSE) 0.354, 22.29%, 0.69<br>- RF (VIM IncNodePurity) 0.262, 16.80%, 0.73<br>- GBM (VIM IncNodePurity) 0.350, 22.87%, 0.69<br>- SVM (kernel function) 0.297, 20.03%, 0.63

Authors	Objective	Formation	Algorithm & Benchmarks	Specifications	Input data	Database	Results
							- cluster 1-RF (VIM IncNodePurity) 0.224, 14.94%, 0.74<br>- cluster 2-RF (VIM IncNodePurity) 0.209, 12.05%, 0.88
(Liao et al., 2020)	To forecast cumulative oil production at the early stage	Cardium Light Tight Oil Play	- RF<br>- MAPE	- 10-fold cross-validation	10 variables	1,286 wells (85% for train and 15% for test)	- MAPE of 15%
(Kong et al., 2021)	To predict the first 12-month cumulative production	Duvernay	- XGBoost, stacked with LR<br>- MSE and R^2	- XGBoost, all hyperparameters are optimized using a Bayesian optimization algorithm<br>- 10-fold cross-validation	22 variables	519 wells (90% for train, 10% for test)	R^2 for train and test:<br>- XGBoost: 0.95, 0.79<br>- Stacked: 0.96, 0.8
(Li et al., 2021, 2020)	To predict the monthly production performance	Permian and Appalachian Basins	- RF, RF-DPR<br>- R^2, MAPE, MADPE	- 10-fold cross-validation	-	over 20,000 horizontal wells	- RF-DPR has higher R^2 and lower MAPE and MADPE, compared with RF and modified Arps DCA<br>- a much smaller error skewness

Authors	Objective	Formation	Algorithm & Benchmarks	Specifications	Input data	Database	Results
							than RF without DPR and modified Arps DCA
(Xue et al., 2021)	To predict the dynamic shale gas production rate	Synthetic Data	- MORC and MORF<br>- MAE, MSE, RMSE, R^2	- MORF<br>N_estimators: 190, N_features: 9 & 10, Max_depth: 18.<br>- MORC<br>N_estimators: 110, N_features: 10, Max_depth: 13.<br>- 10-fold cross-validation	9 & 10 variables	2,000 simulations (1,500 for train and 500 for the test)	88.6% of the data predicted with R^2 values greater than 0.8 using MORF and 9 features while including the initial production, feature will increase the portion of data with R^2 higher than 0.8 to 91% for MORF and 90.6% for MORC
(Bhattacharyya and Vyas, 2022)	EUR prediction for oil wells	Bakken (US)	- RF<br>- R^2	- maximum no. of decision trees: 100<br>- maximum no. of splits at each node: 6<br>- 20 folds	Four clusters of wells consisting of 17 independent parameters	-wells with a production history of more than 96 months (i.e., 8 years)	R^2 for training:<br>- n: 0.9123<br>- τ: 0.9012<br><br>R^2 for test:<br>- n: 0.8737<br>- τ: 0.8589
(Jafarov, 2022)	To predict the DCA parameters and 6- and 12-month	Five fields in the US (Eagle Ford, Haynesville Shale, Bossier Shale,	- MLR, RF<br>- MAE, MSE, R^2	-	21 parameters	- 53 shale gas wells<br>- 80% for training and 20% for test	For predicting DCA parameters, the RF model performed better, while for

Authors	Objective	Formation	Algorithm & Benchmarks	Specifications	Input data	Database	Results
	cumulative gas production	Marcellus, Marcellus-Upper)					the cumulative gas production prediction, the MLR algorithm outperformed the former one.
(Gao et al., 2022)	Gas Production	A tight gas reservoir (AB)	- GB, DT, RF, SVR, ANN -RMSE & MAPE (acc)	-	8 features	- 1071 wells 80% for training, 20% for testing	for GB, DT, RF, SVR, ANN: - RMSE: 21.3, 30.24, 10.9, 25.68, 28.32. - Accuracy (MAPE): 82.08%, 77.23%, 85.25%, 83.99%, 81.20%.
(Johan et al., 2023)	Best 3-month production	23 distinct US basins, with the Gulf Coast, Permian, Williston, Appalachian, and Anadarko Basins (94%)	- RF - R^2	-	12 features	- 75,149 wells	- R^2: 0.712 to 0.863 - Latitude and longitude are the most critical parameters,

S5. Details of Papers with the GBM&SVM-Based Predictive Models

Authors	Objective	Formation	Algorithm & Benchmarks	Specifications	Input data	Database	Results
(Schuetter et al., 2018, 2015)	To predict the first 12 months of cumulative oil production	Wolfcamp (Permian Basin)	- LR, RF, GBM. SVM, KM<br>- MAE and MSE	- 10-fold cross-validation	12 variables	476 wells (80% for train, 20% for test)	MAE and MSE:<br>- LR: 32.39, 2279.71<br>- RF: 25.54, 1272.06<br>- GBM: 24.88, 1227.32<br>- SVM: 25.88, 1503.75<br>- KM: 25.69, 1295.07
(Vyas et al., 2017)	To predict the decline curve parameters for 12 months of production oil production	Eagle Ford	- RF, SVM, MARS<br>- RMSE, MAE, R^2	-	9 variables	80% for train and 20% for test	- SEDM with SVM is found to be more suitable to predict flow rates compared to other models using Machine Learning<br>- The initial flow rate is the most important variable
(Panja et al., 2018)	To predict oil recovery and gas-oil ratio (GOR) from hydraulically fractured low permeability reservoir	Synthetic Data	- ANN, LSSVM, and a second-order polynomial RSM with PSO<br>- NRMSE, R^2	- ANN: 1 hidden layer with 14 neurons<br>- PSO: N_particles for single parameter: 100, C_1: 2, C_2: 2, w: 0.6, and Max_iteration: 1000	8 variables	144 simulations (114 for train and 30 for test)	RSM, LSSVM, ANN<br>Oil Recovery:<br>R^2 (train data): 0.98-0.99, 0.98-0.99, 0.96-0.99<br>NRMSE (train data): 1.9-3.5, 1.7-3.3, 2.1-3.5<br>R^2 (test data): 0.57-0.97, 0.52-0.93, 0.48-0.84,<br>NRMSE (test data): 4.9-20.7, 7.3-21.2, 10.8-22.6<br><br>GOR:

Authors	Objective	Formation	Algorithm & Benchmarks	Specifications	Input data	Database	Results
							R^2 (train data): 0.83-0.98, 0.77-0.99, 0.84-0.97<br>NRMSE (train data): 2.6-5.8, 2-6.8, 3.7-5.6<br>R^2 (test data): 0.41-0.93, 0.45-0.91, 0.3-0.9,<br>NRMSE (test data): 7.9-24, 9.3-18.4, 9.7-26.2
(Shahkarami et al., 2018)	To estimate the dry gas EUR	Marcellus Shale Asset in Southwestern Pennsylvania	- LR, SVM, ANNs, and GP<br>- MSE, RMSE, RAE, RRSE, R^2	-	25 variables	820 horizontal wells	- The results for LR, SVM, ANN, and GP are:<br>- R^2: 0.91, 0.92, 0.86, 0.91<br>- RMSE: 1.98, 1.94, 2.65, 2.04<br>- RAE: 34%, 33%, 42%,35%<br>- MAE: 1.38, 1.33, 1.72, 1.42
(Amr et al., 2018)	To predict the monthly production of unconventional horizontal wells	DJ Basin	- Bayesian regularized neural networks, support vector machine, 2 random forest algorithms, extreme gradient boosting, Stochastic	- 5 folds cross-validation	well location, reservoir properties, drilling and completion data, and neighboring well information	Monthly production history for 713 wells	The most accurate model outperforms Arps'-based estimates by 23% to 36%.

Authors	Objective	Formation	Algorithm & Benchmarks	Specifications	Input data	Database	Results
			Gradient Boosting, generalized linear model, Deep neural networks<br>- Accuracy				
(Baki et al., 2021)	To predict 90-day cumulative BOE	Eagle Ford	- XGBoost, ANN, SVM<br>- RMSE, MAE, R^2	- Cross-validation	10 parameters (8 completion + 2 arial)	7,282 horizontal wells:<br>80% for training and 20% for test	For RMSE, MAE, R^2:<br><br>- XGBoost: 25.331, 16.72, 0.62<br>- ANN: 18.959, 18.96, 0.49<br>- SVM: 28.799, 19.48, 0.51
(Niu et al., 2022)	To predict EUR for shale gas wells	Wei-Yuan, Chang-Ning, Lu-Zhou Blocks, China	- RF, KNN, SVM, GBDT<br>- R^2, MAPE	- Grid search for hyperparameters tuning	Early data of 161 production wells (four schemes considering the importance parameters)	10 early factors affecting the EUR, including the early flowback rate, the cumulative production at different stages of the early stage and the test production<br>- 112 wells for training, 49 wells for testing	For Scheme 3:<br>- R^2 (training set) for RF, KNN, SVM, GBDT: 0.7756 0.7983 0.8124 0.7865<br>- MAPE (test set) for RF, KNN, SVM, GBDT: 17.08% 15.61% 13.41% 19.14%
(Zhai et al., 2022)	To predict the shale gas well production potential	Chang-Ning Field, China	- XGBoost, RF, ANN, SVR<br>- R^2, MAE, MSE, RMSE	- XGBoost: No. of trees: 2000, Max depth: 6, iterative learning rate: 0.5, Min 2nd	14 input features	- 384 production wells<br>- 80% for training and 20% for test	For XGBoost, RF, ANN, SVR:<br><br>- R^2:

Authors	Objective	Formation	Algorithm & Benchmarks	Specifications	Input data	Database	Results
				derivative in the leaf node: 1<br>- RF: 2000 independent decision trees<br>- ANN: RELU, two hidden layers with 14 neurons<br>- SVR: RBF kernel, gamma: 1, and C: 1			train: 0.903, 0.952, 0.788, 0.861<br>test: 0.794, 0.806, 0.716, 0.843<br>- MAE:<br>train: 1.505, 1.023, 0.068, 0.062<br>test: 2.371, 2.371, 0.085, 0.068<br>- MSE:<br>train: 3.938, 2.284, 0.009, 0.006<br>test: 9.903, 9.387, 0.014, 0.007<br>- RMSE:<br>train: 1.984, 1.511, 0.094, 0.077<br>test: 3.147, 3.064, 0.012, 0.084
(Hui et al., 2023)	the gas production	Duvernay	- RF, GBDT, ANN, ET<br>- MSE, R^2	-	- 8 input parameters	- 251 wells 80% training and 20% testing	- The average R^2 of each algorithm using these parameters is 0.77 for ANN, 0.82 for ET, 0.76 for GBDT, and 0.69 for RF.<br>- The average mean-squared errors (MSE) are calculated to be 0.241 for ANN, 0.176 for ET, 0.225 for GBDT, and 0.262 for RF

Authors	Objective	Formation	Algorithm & Benchmarks	Specifications	Input data	Database	Results
(Ren et al., 2023)	3rd year oil production	Permian Basin	- NGB, RF, RMSE<br>- R^2, RMSE	-	geology, geospatial, well spacing, completion, the historical well production and parent-child relationship data	-	Training, validation, and test:<br>- R^2: RF (0.707, 0.625, 0.691), NGB (0.914, 0.714, 0.741), MLP (0.714, 0.681, 0.675)<br>- RMSE: RF (1.869, 2.063, 1.864), NGB (1.000, 1.749, 1.815), MLP (1.821, 1.844, 2.031)

S6. Details of Papers with Other Predictive Models

Authors	Objective	Formation	Algorithm & Benchmarks	Specifications	Input data	Database	Results
(LaFollette et al., 2012)	To evaluate feature influence ranking on the gas production rate	Barnett Shale	- Boosted tree	-	6 variables	16,970 wells (12,043 horizontal and 4,368 vertical)	The most important variables:<br>- true vertical depth<br>- Y path<br>- total fracturing fluid volume<br>- fracturing slurry average stage injection rate<br>- 20/40 mesh proppant
(Gullickson et al., 2014)	To evaluate the Cum oil	Williston Basin Bakken/Three Forks	- GWR and LR- PCA<br>- R^2	-	15 variables	87 wells	R^2 for 180, 360, 540, 720:<br>- GWR: 0.9007, 0.8927, 0.8932, 0.8829<br>- PCR: 0.7460, 0.6526, 0.7262, 0.6693
(Fulford et al., 2016, 2015)	To forecast the 5-year cumulative production and to quantify the completion design impact on rate-time behaviour	Bakken, Cleveland Sand, and Wolfcamp	-	-	-	136 liquid-rich wells in Bakken, 235 tight-oil wells in the Cleveland sand, and 124 liquid-rich wells in Wolfcamp	- the 5-year cumulative-production hindcast from 6% to a range of 4-0.5%, depending on high, medium, or low strength of belief (Bakken)<br>- the method yielded insight into the impact of changing the lateral length and fracture stages and spacing upon time/rate behaviour (the

Authors	Objective	Formation	Algorithm & Benchmarks	Specifications	Input data	Database	Results
							Cleveland sand formation)<br>- fracture conductivity did not increase ultimate recovery; rather, it accelerated production (Wolfcamp)
(Mohagheg h, 2016)	To assess the influential parameters on well productivit y	Marcellus shale	- Fuzzy set<br>- Classification	-	9 variables	221 wells	In low-quality shale, the completion design parameters are not as important as those in high-quality shale
(Schuetter et al., 2019)	To predict the cumulative oil production within the first year of operation	Wolfcamp (Permian Basin)	- LR, QPR, RF, SVM, GBM, and Ensembles (direct averaging-M1, Weighted average-M2, stacking, NN-M3a, and RF-M3b)<br>- RMSE and PRMSE	- 10-fold cross-validation	12 variables	318 horizontal wells	RMSE and PRMSE (k bbl) for 1st 12 months cum oil:<br>Ensemble methods<br>- M1: 37.57, 4.197<br>- M2: 37.45, 4.426<br>- M3a: 36.21, 4.839<br>- M3b: 36.15, 8.961<br>Constituent<br>- LR: 47.12, 5.031<br>- QPR: 40.03, 8.184<br>- SVM: 39, 4.683<br>- RF: 38.33, 3.056<br>- GBM: 40.40, 13.404
(Kong et al., 2020)	To simulate the gas well performance with a very short	Montney	- MCMC, Bootstrap, and Deterministic Regression	-	- 344 gas wells	- Monthly gas production history	Ranges of RMSE, SMAPE, and MAPE for 3 cases:<br>- PDR-DCA: 295-31317, 6-41, 0.11-0.52

Authors	Objective	Formation	Algorithm & Benchmarks	Specifications	Input data	Database	Results
	production history		- RMSE, SMAPE, MAPE				- Bootstrap: 294-28313, 8-48, 0.15-0.86<br>- Deterministic Regression: 302-28635, 8-47, 0.14-0.82
(Tadjer et al., 2021)	To demonstrate a method of machine learning that could replace or accelerate manual DCA for short-term oil well forecasting	Midland Field	- DeepAR and Prophet model<br>- mean CRPS	DeepAR:<br>- epochs 100<br>- batch size 32<br>- batches/epoch 100<br>- context length: 24<br>- Layers: 1,2,4<br>Cell type: GRU, LSTM<br>Cell hidden state size: 1, 54, 560<br>Gaussian number: 1, 3, 8<br>Dropout rate: 0.1, 0.4, 0.6<br>Learning rate: 1e-4, 1e-3, 1e-2	Production time series	22 wells with 105 to 362 months (the last 24 months for test)	The results showed that the deep learning approach and Prophet analysis yield a satisfactory result in short-term forecasts. Still, they may fail to identify long-term trends in predictions unless the predictions are constantly adjusted.
(Hui et al., 2021)	To forecast the 12-month shale gas production	Duvernay	LR, ANN, extra tree, GBM<br>- MSE and R^2	-	13 variables	573 horizontal wells (80% for train and 20% for test)	R^2 and MSE:<br>Train:<br>- LR: 0.67, 0.033<br>- ANN: 0.813, 0.021<br>- Extra trees: 0.971, 0.029<br>- GBM: 0.972, 0.028<br>Test:<br>- LR: 0.653, 0.342<br>- ANN: 0.729, 0.301

Authors	Objective	Formation	Algorithm & Benchmarks	Specifications	Input data	Database	Results
							- Extra trees: 0.809, 0.194<br>- GBM: 0.794, 0.205
(Meng et al., 2022)	To analyze shale gas production performance (EUR)	Fuling Shale Gas Field (China)	- RF, GBDT, and XGBoost, Ensembles<br>- PSO, DE, and BO<br>- R^2, MSE, MAE	- Linear meta model	13 independent variables	- 254 shale gas wells<br>- 80% for training and 20% for test	RF, GBDT, XGBoost, Stacked:<br><br>- R^2:<br>train: 0.747, 0.858, 0.810, 0.791<br>test: 0.652, 0.703, 0.682, 0.743<br>- MSE:<br>train: 0.160, 0.090, 0.120, 0.132<br>test: 0.218, 0.187, 0.202, 0.161<br>- MAE:<br>train: 0.317, 0.221, 0.256, 0.276<br>test: 0.371, 0.332, 0.342, 0.308

Reference

Akbilgic, O., Zhu, D., Gates, I.D., Bergerson, J.A., 2015. Prediction of steam-assisted gravity drainage steam to oil ratio from reservoir characteristics. Energy 93, 1663–1670. https://doi.org/10.1016/j.energy.2015.09.029

Alabboodi, M.J., Mohaghegh, S.D., 2016. Conditioning the estimating ultimate recovery of shale wells to reservoir and completion parameters, in: SPE Eastern Regional Meeting. OnePetro. https://doi.org/10.2118/184064-MS

Alimohammadi, H., Rahmanifard, H., Chen, N., 2020. Multivariate time series modelling approach for production forecasting in unconventional resources, in: Proceedings - SPE Annual Technical Conference and Exhibition. OnePetro. https://doi.org/10.2118/201571-ms

Amirian, E., Leung, J.Y., Zanon, S., Dzurman, P., 2015. Integrated cluster analysis and artificial neural network modeling for steam-assisted gravity drainage performance prediction in heterogeneous reservoirs. Expert Syst Appl 42, 723–740. https://doi.org/10.1016/j.eswa.2014.08.034

Amr, S., El Ashhab, H., El-Saban, M., Schietinger, P., Caile, C., Kaheel, A., Rodriguez, L., 2018. A large-scale study for a multi-basin machine learning model predicting horizontal well production, in: Proceedings - SPE Annual Technical Conference and Exhibition. OnePetro. https://doi.org/10.2118/191538-ms

Apaydin, O.G.. G., Ozkan, E.., Raghavan, R.., 2012. Effect of discontinuous microfractures on ultratight matrix permeability of a dual-porosity medium, in: SPE Reservoir Evaluation and Engineering. OnePetro, pp. 473–485. https://doi.org/10.2118/147391-PA

Baki, S., Temizel, C., Dursun, S., 2021. Well completion optimization in unconventional reservoirs using machine learning methods, in: Proceedings - SPE Annual Technical Conference and Exhibition. OnePetro. https://doi.org/10.2118/206241-MS

Behmanesh, H.., Mattar, L.., Thompson, J.M., Anderson, D.M., Nakaska, D.W., Clarkson, C.R., 2018. Treatment of rate-transient analysis during boundary-dominated flow. SPE Journal 23, 1145–1165. https://doi.org/10.2118/189967-pa

Bhattacharyya, S., Vyas, A., 2022. Application of machine learning in predicting oil rate decline for Bakken shale oil wells. Sci Rep 12, 1–18. https://doi.org/10.1038/s41598-022-20401-6

Bowie, B., 2018. Machine learning applied to optimize duvernay well performance, in: Society of Petroleum Engineers - SPE Canada Unconventional Resources Conference, URC 2018. OnePetro. https://doi.org/10.2118/189823-ms

Breiman, L., 2001. Random forests. Mach Learn 45, 5–32. https://doi.org/10.1023/A:1010933404324/METRICS

Brownlee, J., 2021. A Gentle Introduction to Ensemble Learning Algorithms. MachineLearningMastery.com. URL https://machinelearningmastery.com/tour-of-ensemble-learning-algorithms/ (accessed 10.2.23).

Brownlee, J., 2020. A Gentle Introduction to the Gradient Boosting Algorithm for Machine Learning. MachineLearningMastery.com. URL https://machinelearningmastery.com/gentle-introduction-gradient-boosting-algorithm-machine-learning/ (accessed 11.11.23).

Brownlee, J., 2018. Deep Learning for Time Series Forecasting Predict the Future with MLPs, CNNs and LSTMs in Python. URL https://machinelearningmastery.com/deep-learning-for-time-series-forecasting/

Brownlee, J., 2017. Master Machine Learning Algorithms. Machine learning mastery. URL https://machinelearningmastery.com/master-machine-learning-algorithms/ (accessed 1.17.22).

Cao, Q.., Banerjee, R.., Gupta, S.., Li, J.., Zhou, W.., Jeyachandra, B.., 2016. Data driven production forecasting using machine learning, in: Society of Petroleum Engineers - SPE Argentina Exploration and Production of Unconventional Resources Symposium. OnePetro. https://doi.org/10.2118/180984-ms

Chaikine, I., 2020. Machine Learning Applications for Production Prediction and Optimization in Multistage Hydraulically Fractured Wells. URL https://prism.ucalgary.ca/bitstream/handle/1880/112817/ucalgary_2020_chaikine_ilia.pdf?sequence=2&isAllowed=y (accessed 8.6.21).

Chen, T., Guestrin, C., 2016. XGBoost: A Scalable Tree Boosting System. Proceedings of the ACM SIGKDD International Conference on Knowledge Discovery and Data Mining 13-17-August-2016, 785–794. https://doi.org/10.1145/2939672.2939785

Chen, X., Li, J., Gao, P., Zhou, J., 2022. Prediction of shale gas horizontal wells productivity after volume fracturing using machine learning–an LSTM approach. Pet Sci Technol 40, 1861–1877. https://doi.org/10.1080/10916466.2022.2032739

Clark, A.J., 2011. Decline curve analysis in unconventional resource plays using logistic growth models. URL https://repositories.lib.utexas.edu/handle/2152/ETD-UT-2011-08-4201 (accessed 2.18.23).

Clarkson, C.R., 2013. Production data analysis of unconventional gas wells: Review of theory and best practices. Int J Coal Geol. https://doi.org/10.1016/j.coal.2013.01.002

Clarkson, C.R., Jordan, C.L., Ilk, D., Blasingame, T.A., 2012. Rate-transient analysis of 2-phase (gas + water) CBM wells. J Nat Gas Sci Eng 8, 106–120. https://doi.org/10.1016/j.jngse.2012.01.006

Crnkovic-Friis, L.., Erlandson, M.., 2015. Geology driven EUR prediction using deep learning, in: Proceedings - SPE Annual Technical Conference and Exhibition. OnePetro, pp. 1062–1071. https://doi.org/10.2118/174799-ms

Dobilas, S., 2020. Support Vector Regression (SVR) — One of the Most Flexible Yet Robust Prediction Algorithms. URL https://towardsdatascience.com/support-vector-regression-svr-one-of-the-most-flexible-yet-robust-prediction-algorithms-4d25fbdaca60 (accessed 1.18.22).

Duong, A.N., 2010. An Unconventional Rate Decline Approach for Tight and Fracture-Dominated Gas Wells. Society of Petroleum Engineers - Canadian Unconventional Resources and International Petroleum Conference 2010 3, 2052–2066. https://doi.org/10.2118/137748-MS

Ebden, M., 2008. Gaussian Processes: A Quick Introduction. URL http://arxiv.org/abs/1505.02965 (accessed 1.18.22).

EDUCBA, 2020. Decision Tree in Machine Learning | Split creation and Building a Tree. URL https://www.educba.com/decision-tree-in-machine-learning/ (accessed 1.18.22).

Enyioha, C., Ertekin, T., 2017. Performance prediction for advanced well structures in unconventional oil and gas reservoirs using artificial intelligent expert systems, in: Proceedings - SPE Annual Technical Conference and Exhibition. OnePetro. https://doi.org/10.2118/187037-ms

Enyioha, C., Ertekin, T., 2014. Advanced well structures: An artificial intelligence approach to field deployment and performance prediction, in: Society of Petroleum Engineers - SPE

Intelligent Energy International 2014. OnePetro, pp. 549–561. https://doi.org/10.2118/167870-ms

Esmaili, S.., Kalantari-Dahaghi, A.., Mohaghegh, S.D., 2012a. Forecasting, sensitivity and economic analysis of hydrocarbon production from Shale plays using artificial intelligence & data mining, in: Society of Petroleum Engineers - SPE Canadian Unconventional Resources Conference 2012, CURC 2012. OnePetro, pp. 922–930. https://doi.org/10.2118/162700-ms

Esmaili, S.., Kalantari-Dahaghi, A.., Mohaghegh, S.D., 2012b. Modeling and history matching of hydrocarbon production from Marcellus shale using data mining and pattern recognition technologies, in: SPE Eastern Regional Meeting. OnePetro, pp. 143–154. https://doi.org/10.2118/161184-ms

Esmaili, S., Mohaghegh, S.D., 2016. Full field reservoir modeling of shale assets using advanced data-driven analytics. Geoscience Frontiers 7, 11–20. https://doi.org/10.1016/j.gsf.2014.12.006

Fan, D., Sun, H., Yao, J., Zhang, K., Yan, X., Sun, Z., 2021. Well production forecasting based on ARIMA-LSTM model considering manual operations. Energy 220, 119708. https://doi.org/10.1016/j.energy.2020.119708

Fulford, D.S., Bowie, B., Berry, M.E., Bowen, B., Turk, D.W., 2016. Machine learning as a reliable technology for evaluating time/rate performance of unconventional wells. SPE Economics and Management 8, 23–39. https://doi.org/10.2118/174784-PA

Fulford, D.S., Bowie, B., Berry, M.E., Bowen, B., Turk, D.W., 2015. Machine learning as a reliable technology for evaluating time-rate performance of unconventional wells, in: Proceedings - SPE Annual Technical Conference and Exhibition. OnePetro, pp. 856–886. https://doi.org/10.2118/174784-ms

Gao, Q., Liao, L., Yang, S., 2022. Application of artificial intelligence technology in unconventional natural gas production forecasting. SPIE 291. https://doi.org/10.1117/12.2662821

Geurts, P., Ernst, D., Wehenkel, L., 2006. Extremely randomized trees. Mach Learn 63, 3–42. https://doi.org/10.1007/s10994-006-6226-1

Ghoshal, A., 2020. Types of Neural Networks. EDUCBA. URL https://www.educba.com/types-of-neural-networks/

Goyal, K., 2020. Machine Learning vs Neural Networks: What is the Difference?. upGrad blog. URL https://www.upgrad.com/blog/machine-learning-vs-neural-networks/ (accessed 1.18.22).

Grujic, O., Da Silva, C., Caers, J., 2015. Functional approach to data mining, forecasting, and uncertainty quantification in unconventional reservoirs, in: Proceedings - SPE Annual Technical Conference and Exhibition. OnePetro, pp. 1704–1720. https://doi.org/10.2118/174849-ms

Gullickson, G., Fiscus, K., Cook, P., 2014. Completion influence on production decline in the bakken/three forks play, in: Society of Petroleum Engineers - SPE Western North American and Rocky Mountain Joint Meeting. OnePetro. https://doi.org/10.2118/169531-ms

Gupta, I., Rai, C., Sondergeld, C., Devegowda, D., 2018. Variable Exponential Decline: Modified Arps To Characterize Unconventional-Shale Production Performance. SPE Reservoir Evaluation & Engineering 21, 1045–1057. https://doi.org/10.2118/194005-PA

Han, D., Jung, J., Kwon, S., 2020. Comparative study on supervised learning models for productivity forecasting of shale reservoirs based on a data-driven approach. Applied Sciences (Switzerland) 10. https://doi.org/10.3390/app10041267

Han, D., Kwon, S., Son, H., Lee, J., 2019. Production forecasting for shale gas well in transient flow using machine learning and decline curve analysis, in: SPE/AAPG/SEG Asia Pacific Unconventional Resources Technology Conference 2019, APUR 2019. OnePetro. https://doi.org/10.15530/ap-urtec-2019-198198

Haskett, W.J., Brown, P.J., 2005. Evaluation of Unconventional Resource Plays. SPE Hydrocarbon Economics and Evaluation Symposium 1–11. https://doi.org/10.2118/96879-MS

Hui, G., Chen, S., He, Y., Wang, H., Gu, F., 2021. Machine learning-based production forecast for shale gas in unconventional reservoirs via integration of geological and operational factors. J Nat Gas Sci Eng 94, 104045. https://doi.org/10.1016/j.jngse.2021.104045

Hui, G., Chen, Z., Wang, Y., Zhang, D., Gu, F., 2023. An integrated machine learning-based approach to identifying controlling factors of unconventional shale productivity. Energy 266, 126512. https://doi.org/10.1016/J.ENERGY.2022.126512

Ibrahim, M.H., Wattenbarger, R.A., 2006. Analysis of Rate Dependence in Transient Linear Flow in Tight Gas Wells. OnePetro. https://doi.org/10.2118/100836-ms

Ilk, D., Rushing, J.A., Perego, A.D., Blasingame, T.A., 2008. Exponential vs. Hyperbolic Decline in Tight Gas Sands — Understanding the Origin and Implications for Reserve Estimates Using Arps' Decline Curves. Proceedings - SPE Annual Technical Conference and Exhibition 7, 4637–4659. https://doi.org/10.2118/116731-MS

Jafarov, A., 2022. Investigation of the Relationship between Operational Parameters and Decline Curve Characteristics in Shale Gas Wells using Data Analytics and Machine Learning (Master Thesis). Politecnico di Torino.

Johan, D.C., Shukla, P., Shrivastava, K., Koley, M., 2023. Data-Driven Completion Optimization for Unconventional Assets. Proceedings of the 11th Unconventional Resources Technology Conference. https://doi.org/10.15530/URTEC-2023-3861032

Kaggle, 2020. A Guide on XGBoost hyperparameters tuning. URL https://www.kaggle.com/code/prashant111/a-guide-on-xgboost-hyperparameters-tuning/notebook (accessed 4.13.22).

Khanal, A., Khoshghadam, M., Lee, W.J., Nikolaou, M., 2017. New forecasting method for liquid rich shale gas condensate reservoirs with data driven approach using principal component analysis. J Nat Gas Sci Eng 38, 621–637. https://doi.org/10.1016/j.jngse.2017.01.014

Kong, B., Chen, S., Chen, Z., Zhou, Q., 2020. Bayesian probabilistic dual-flow-regime decline curve analysis for complex production profile evaluation. J Pet Sci Eng 195, 107623. https://doi.org/10.1016/j.petrol.2020.107623

Kong, B., Chen, Z., Chen, S., Qin, T., 2021. Machine learning-assisted production data analysis in liquid-rich Duvernay Formation. J Pet Sci Eng 200, 108377. https://doi.org/10.1016/j.petrol.2021.108377

LaFollette, R.F., Holcomb, W.D., Aragon, J., 2012. Practical data mining: Analysis of Barnett Shale production results with emphasis on well completion and fracture stimulation, in: Society of Petroleum Engineers - SPE Hydraulic Fracturing Technology Conference 2012. OnePetro, pp. 591–600. https://doi.org/10.2118/152531-ms

Lee, K., Lim, J., Yoon, D., Jung, H., 2019. Prediction of shale-gas production at duvernay formation using deep-learning algorithm. SPE Journal 24, 2423–2437. https://doi.org/10.2118/195698-PA

Lee, K.J., 2020. Data-Driven models to predict hydrocarbon production from unconventional reservoirs by thermal recovery. Journal of Energy Resources Technology, Transactions of the ASME 142. https://doi.org/10.1115/1.4047309

Li, B., Billiter, T., Tokar, T., 2020. Significant Error Reduction in Machine-Learning Decline Curve Analysis for Unconventional Reservoirs. OnePetro. https://doi.org/10.15530/urtec-2020-2106

Li, B., Billiter, T.C., Tokar, T., 2021. Rescaling Method for Improved Machine-Learning Decline Curve Analysis for Unconventional Reservoirs. SPE Journal 26, 1759–1772. https://doi.org/10.2118/205349-pa

Li, D., You, S., Liao, Q., Sheng, M., Tian, S., 2023. Prediction of Shale Gas Production by Hydraulic Fracturing in Changning Area Using Machine Learning Algorithms. Transp Porous Media 149, 373–388. https://doi.org/10.1007/s11242-023-01935-3

Li, W., Dong, Z., Lee, J.W., Ma, X., Qian, S., 2022. Development of Decline Curve Analysis Parameters for Tight Oil Wells Using a Machine Learning Algorithm. Geofluids 2022. https://doi.org/10.1155/2022/8441075

Li, Y., Han, Y., 2017. Decline curve analysis for production forecasting based on machine learning, in: Society of Petroleum Engineers - SPE Symposium: Production Enhancement and Cost Optimisation 2017. OnePetro. https://doi.org/10.2118/189205-ms

Liang, Y., Zhao, P., 2019. A machine learning analysis based on big data for eagle ford shale formation, in: Proceedings - SPE Annual Technical Conference and Exhibition. OnePetro. https://doi.org/10.2118/196158-ms

Liao, L., Zeng, Y., Liang, Y., Zhang, H., 2020. Data mining: A novel strategy for production forecast in tight hydrocarbon resource in Canada by random forest analysis, in: International

Petroleum Technology Conference 2020, IPTC 2020. OnePetro. https://doi.org/10.2523/iptc-20344-ms

Lolon, E.., Hamidieh, K.., Weijers, L.., Mayerhofer, M.., Melcher, H.., Oduba, O.., 2016. Evaluating the relationship between well parameters and production using multivariate statistical models: A middle Bakken and three forks case history, in: Society of Petroleum Engineers - SPE Hydraulic Fracturing Technology Conference, HFTC 2016. OnePetro. https://doi.org/10.2118/179171-ms

López-Flores, F.J., Lira-Barragán, L.F., Rubio-Castro, E., El-Halwagi, M.M., Ponce-Ortega, J.M., 2023. Hybrid Machine Learning-Mathematical Programming Approach for Optimizing Gas Production and Water Management in Shale Gas Fields. ACS Sustain Chem Eng 11, 6043–6056. https://doi.org/10.1021/acssuschemeng.3c00569

Lu, C., Jiang, H., Yang, J., Wang, Z., Zhang, M., Li, J., 2022. Shale oil production prediction and fracturing optimization based on machine learning. J Pet Sci Eng 217, 110900. https://doi.org/10.1016/j.petrol.2022.110900

Luo, G., Tian, Y., Bychina, M., Ehlig-Economides, C., 2018. Production optimization using machine learning in bakken shale, in: SPE/AAPG/SEG Unconventional Resources Technology Conference 2018, URTC 2018. OnePetro. https://doi.org/10.15530/urtec-2018-2902505

Luo, G., Tian, Y., Sharma, A., Ehlig-Economides, C., 2019. Eagle ford well insights using data-driven approaches, in: International Petroleum Technology Conference 2019, IPTC 2019. OnePetro. https://doi.org/10.2523/iptc-19260-ms

Mathworks, 2020. Choose Regression Model Options: MATLAB & Simulink. Mathworks.com. URL https://www.mathworks.com/help/stats/choose-regression-model-options.html#bvi2d8a-19 (accessed 8.30.21).

Meng, J., Zhou, Y.-J., Ye, T.-R., Xiao, Y.-T., Lu, Y.-Q., Zheng, A.-W., Liang, B., 2022. Hybrid data-driven framework for shale gas production performance analysis via game theory, machine learning, and optimization approaches. Pet Sci. https://doi.org/10.1016/j.petsci.2022.09.003

Michel, G.., Sigal, R.., Civan, F.., Devegowda, D.., 2012. Effect of capillary relaxation on water entrapment after hydraulic fracturing stimulation, in: Society of Petroleum Engineers - SPE Americas Unconventional Resources Conference 2012. OnePetro, pp. 583–596. https://doi.org/10.2118/155787-ms

Mitchell, T.M., 2010. Machine Learning, Machine Learning. Machine Learning V2. https://doi.org/10.1093/bioinformatics/btq112

ML Glossary, 2017. Gradient Descent — ML Glossary documentation. URL https://ml-cheatsheet.readthedocs.io/en/latest/gradient_descent.html (accessed 9.9.21).

Mohaghegh, S.D., 2017. Shale analytics: Data-driven analytics in unconventional resources, Shale Analytics: Data-Driven Analytics in Unconventional Resources. https://doi.org/10.1007/978-3-319-48753-3

Mohaghegh, S.D., 2016. Determining the main drivers in hydrocarbon production from shale using advanced data-driven analytics – A case study in Marcellus shale. Journal of Unconventional Oil and Gas Resources 15, 146–157. https://doi.org/10.1016/J.JUOGR.2016.07.004

Mohaghegh, S.D., Gaskari, R., Maysami, M., 2017. Shale analytics: Making production and operational decisions based on facts: A case study in marcellus shale, in: Society of Petroleum

Engineers - SPE Hydraulic Fracturing Technology Conference and Exhibition 2017. OnePetro, pp. 141–163. https://doi.org/10.2118/184822-ms

Mohammadmoradi, P., Moradi, H.M., Kantzas, A., 2018. Data-driven production forecasting of unconventional wells with apache spark, in: SPE Western Regional Meeting Proceedings. OnePetro. https://doi.org/10.2118/190098-ms

Mohr, F.X., 2018. An Introduction to Ordinary Least Squares (OLS) in R. r-econometrics. URL https://www.r-econometrics.com/methods/ols/ (accessed 9.9.21).

Nagpal, A., 2017. Decision Tree Ensembles- Bagging and Boosting. Towards Data Science. URL https://towardsdatascience.com/decision-tree-ensembles-bagging-and-boosting-266a8ba60fd9 (accessed 9.9.21).

Nejad, A.M., Sheludko, S., Hodgson, T., McFall, R., Shelley, R.F., 2015. A case history: Evaluating well completions in the Eagle Ford Shale using a data-driven approach, in: Society of Petroleum Engineers - SPE Hydraulic Fracturing Technology Conference 2015. OnePetro, pp. 164–182. https://doi.org/10.2118/spe-173336-ms

Ning, Y., Kazemi, H., Tahmasebi, P., 2022. A comparative machine learning study for time series oil production forecasting: ARIMA, LSTM, and Prophet. Comput Geosci 164, 105126. https://doi.org/10.1016/j.cageo.2022.105126

Niu, W., Lu, J., Sun, Y., 2022. Development of shale gas production prediction models based on machine learning using early data. Energy Reports 8, 1229–1237. https://doi.org/10.1016/j.egyr.2021.12.040

Nobakht, M., Clarkson, C.R., Kaviani, D., 2012. New and improved methods for performing rate-transient analysis of shale gas reservoirs, in: SPE Reservoir Evaluation and Engineering. pp. 335–350. https://doi.org/10.2118/147869-PA

Otero, A., Carballido, J.L., Salgado, L., Canudo, J.I., Garrido, C., Kecerdasan, I., Ikep, P., 2017. Random Forest: Many are Better than One. QuantDare. URL https://quantdare.com/random-forest-many-are-better-than-one/ (accessed 1.17.22).

Ozkan, E.., Raghavan, R.., Apaydin, O.G., 2010. Modeling of fluid transfer from shale matrix to fracture network, in: Proceedings - SPE Annual Technical Conference and Exhibition. OnePetro, pp. 3314–3331. https://doi.org/10.2118/134830-ms

Panja, P., Velasco, R., Pathak, M., Deo, M., 2018. Application of artificial intelligence to forecast hydrocarbon production from shales. Petroleum 4, 75–89. https://doi.org/10.1016/j.petlm.2017.11.003

Park, J., Datta-Gupta, A., Singh, A., Sankaran, S., 2021. Hybrid physics and data-driven modeling for unconventional field development and its application to US onshore basin. J Pet Sci Eng 206, 109008. https://doi.org/10.1016/j.petrol.2021.109008

Pedamkar, P., 2020. Machine Learning Techniques | Top 4 Techniques of Machine Learning. EDUCBA. URL https://www.educba.com/machine-learning-techniques/?source=leftnav (accessed 9.9.21).

Qanbari, F., Clarkson, C.R., 2016. Rate-transient analysis of liquid-rich tight/shale reservoirs using the dynamic drainage area concept: Examples from North American reservoirs. J Nat Gas Sci Eng 35, 224–236. https://doi.org/10.1016/j.jngse.2016.08.049

Qiu, K., Li, J., Chen, D., 2022. Optimized long short-term memory (LSTM) network for performance prediction in unconventional reservoirs. Energy Reports 8, 15436–15445. https://doi.org/10.1016/J.EGYR.2022.11.130

Rahmanifard, H., Alimohammadi, H., Gates, I., 2020. Well Performance Prediction in Montney Formation Using Machine Learning Approaches. OnePetro. https://doi.org/10.15530/urtec-2020-2465

Rahmanifard, H., Gates, I., Shabib-Asl, A., 2022. Comparison of Machine Learning and Statistical Predictive Models for Production Time Series Forecasting in Tight Oil Reservoirs. OnePetro. https://doi.org/10.15530/urtec-2022-3703284

Rahmanifard, H., Plaksina, T., 2019. Application of artificial intelligence techniques in the petroleum industry: a review. Artif Intell Rev. https://doi.org/10.1007/s10462-018-9612-8

Rahmanifard, H., Plaksina, T., 2018. Application of fast analytical approach and AI optimization techniques to hydraulic fracture stage placement in shale gas reservoirs. J Nat Gas Sci Eng 52, 367–378. https://doi.org/10.1016/j.jngse.2018.01.047

Ren, X. (Rex), Yin, J., Xiao, F., Miao, S., Lolla, S., Yao, C., Lonnes, S., Sun, H., Chen, Y., Brown, J.S., Garzon, J., Pankaj, P., 2023. Data Driven Oil Production Prediction and Uncertainty Quantification for Unconventional Asset Development Planning Through Machine Learning. Proceedings of the 11th Unconventional Resources Technology Conference. https://doi.org/10.15530/URTEC-2023-3865670

Schuetter, J., Mishra, S., Lin, L., Chandramohan, D., 2019. Ensemble Learning: A Robust Paradigm for Data-Driven Modeling in Unconventional Reservoirs. SPE/AAPG/SEG Unconventional Resources Technology Conference 2019, URTeC 2019. https://doi.org/10.15530/URTEC-2019-929

Schuetter, J., Mishra, S., Zhong, M., LaFollette, R., 2018. A data-analytics tutorial: Building predictive models for oil production in an unconventional shale reservoir, in: SPE Journal. OnePetro, pp. 1075–1089. https://doi.org/10.2118/189969-pa

Schuetter, J., Mishra, S., Zhong, M., LaFollette, R., 2015. Data Analytics for Production Optimization in Unconventional Reservoirs. https://doi.org/10.15530/URTEC-2015-2167005

scikit-learn, 2020. Ensemble methods. URL https://scikit-learn.org/stable/modules/ensemble.html (accessed 9.9.21).

Shahkarami, A., Ayers, K., Wang, G., Ayers, A., 2018. Application of machine learning algorithms for optimizing future production in Marcellus shale, case study of Southwestern Pennsylvania, in: SPE Eastern Regional Meeting. Society of Petroleum Engineers (SPE). https://doi.org/10.2118/191827-18erm-ms

Shelley, R., Guliyev, N., Nejad, A., 2012a. A novel method to optimize horizontal bakken completions in a factory mode development program, in: Proceedings - SPE Annual Technical Conference and Exhibition. OnePetro, pp. 3034–3043. https://doi.org/10.2118/159696-ms

Shelley, R., Nejad, A., Guliyev, N., Raleigh, M., Matz, D., 2014. Understanding multi-fractured horizontal marcellus completions, in: SPE Eastern Regional Meeting. OnePetro, pp. 18–31. https://doi.org/10.2118/171003-ms

Shelley, R., Saugier, L., Al-Tailji, W., Guliyev, N., Shah, K., 2012b. Understanding hydraulic fracture stimulated horizontal eagle ford completions, in: Society of Petroleum Engineers - SPE Asia Pacific Oil and Gas Conference and Exhibition 2012, APOGCE 2012. OnePetro, pp. 51–61. https://doi.org/10.2118/152121-MS

Smets, K., Verdonk, B., Jordaan, E.M., 2007. Evaluation of performance measures for SVR hyperparameter selection, in: IEEE International Conference on Neural Networks - Conference Proceedings. pp. 637–642. https://doi.org/10.1109/IJCNN.2007.4371031

Smola, A.J., Sch¨olkopf, B., Sch¨olkopf, S., 2004. A tutorial on support vector regression. Stat Comput. URL https://link.springer.com/article/10.1023/B:STCO.0000035301.49549.88 (accessed 10.2.23).

Song, X., Liu, Y., Xue, L., Wang, J., Zhang, J., Wang, J., Jiang, L., Cheng, Z., 2020. Time-series well performance prediction based on Long Short-Term Memory (LSTM) neural network model. J Pet Sci Eng 186, 106682. https://doi.org/10.1016/j.petrol.2019.106682

Suhag, A., Ranjith, R., Aminzadeh, F., 2017. Comparison of shale oil production forecasting using empirical methods and artificial neural networks, in: Proceedings - SPE Annual Technical Conference and Exhibition. OnePetro. https://doi.org/10.2118/187112-ms

Sun, J.., Ma, X.., Kazi, M.., 2018. Comparison of decline curve analysis DCA with recursive neural networks RNN for production forecast of multiple wells, in: SPE Western Regional Meeting Proceedings. OnePetro. https://doi.org/10.2118/190104-ms

Tadjer, A., Hong, A., Bratvold, R.B., 2021. Machine learning based decline curve analysis for short-term oil production forecast. Energy Exploration & Exploitation 014459872110117. https://doi.org/10.1177/01445987211011784

Temizel, C., Canbaz, C.H., Alsaheib, H., Yanidis, K., Balaji, K., Alsulaiman, N., Basri, M., Jama, N., 2021. Geology-Driven EUR Forecasting in Unconventional Fields. SPE Middle East Oil and Gas Show and Conference, MEOS, Proceedings 2021-November. https://doi.org/10.2118/204583-MS

Thavarajah, R., Darabi, H., Ruta, N., Salehi, A., Kianinejad, A., 2022. A Deep Learning Framework for Multi-Horizon Probabilistic Production Forecasting in Unconventional Reservoirs. OnePetro. https://doi.org/10.15530/urtec-2022-3723682

Valkó, P.P., 2009. Assigning value to stimulation in the Barnett Shale: a simultaneous analysis of 7000 plus production hystories and well completion records. Society of Petroleum Engineers - SPE Hydraulic Fracturing Technology Conference 2009 386–404. https://doi.org/10.2118/119369-MS

Valkó, P.P., Lee, W.J., 2010. A Better Way to Forecast Production from Unconventional Gas Wells. Proceedings - SPE Annual Technical Conference and Exhibition 3, 1860–1875. https://doi.org/10.2118/134231-MS

Vikara, D., Khanna, V., 2022. Application of a Deep Learning Network for Joint Prediction of Associated Fluid Production in Unconventional Hydrocarbon Development. Processes 10, 740. https://doi.org/10.3390/pr10040740

Vyas, A., Datta-Gupta, A., Mishra, S., 2017. Modeling early time rate decline in unconventional reservoirs using machine learning techniques, in: Society of Petroleum Engineers - SPE Abu Dhabi International Petroleum Exhibition and Conference 2017. OnePetro. https://doi.org/10.2118/188231-ms

Wang, K., Li, H., Wang, J., Jiang, B., Bu, C., Zhang, Q., Luo, W., 2017. Predicting production and estimated ultimate recoveries for shale gas wells: A new methodology approach. Appl Energy 206, 1416–1431. https://doi.org/10.1016/J.APENERGY.2017.09.119

Wang, S., Chen, S., 2019. Insights to fracture stimulation design in unconventional reservoirs based on machine learning modeling. J Pet Sci Eng 174, 682–695. https://doi.org/10.1016/j.petrol.2018.11.076

Wang, S., Chen, S., 2016. A comprehensive evaluation of well completion and production performance in Bakken shale using data-driven approaches, in: Society of Petroleum

Engineers - SPE Asia Pacific Hydraulic Fracturing Conference. OnePetro. https://doi.org/10.2118/181803-ms

Wang, S., Chen, Z., Chen, S., 2019. Applicability of deep neural networks on production forecasting in Bakken shale reservoirs. J Pet Sci Eng 179, 112–125. https://doi.org/10.1016/j.petrol.2019.04.016

Werbos, P.J., 2004. What is a neural network?, in: Handbook of Neural Computation. https://doi.org/10.1887/0750303123/b365c4

Williams, B., Weaver, B., Weijers, L., 2015. Completing the Second Target: Finding Optimal Completion Practices for the Three Forks Formation in the Williston Basin Using Multivariate Statistical Analysis. Society of Petroleum Engineers (SPE). https://doi.org/10.2118/spe-173377-ms

Xue, L., Liu, Yuetian, Xiong, Y., Liu, Yanli, Cui, X., Lei, G., 2021. A data-driven shale gas production forecasting method based on the multi-objective random forest regression. J Pet Sci Eng 196, 107801. https://doi.org/10.1016/j.petrol.2020.107801

Zhai, S., Geng, S., Li, C., Gong, Y., Jing, M., Li, Y., 2022. Prediction of gas production potential based on machine learning in shale gas field: a case study. Energy Sources, Part A: Recovery, Utilization and Environmental Effects 44, 6581–6601. https://doi.org/10.1080/15567036.2022.2100521

Zhan, C., Sankaran, S., LeMoine, V., Graybill, J., Mey, D.O.S., 2020. Application of machine learning for production forecasting for unconventional resources, in: SPE/AAPG/SEG Unconventional Resources Technology Conference 2020, URTeC 2020. OnePetro. https://doi.org/10.15530/urtec-2019-47

Zhang, H., Cocco, M., Rietz, D., Cagle, A., Lee, J., 2015. An Empirical Extended Exponential Decline Curve for Shale Reservoirs. Proceedings - SPE Annual Technical Conference and Exhibition 2015-January, 4118–4140. https://doi.org/10.2118/175016-MS

Zhong, M., Schuetter, J., Mishra, S., LaFollette, R.F., 2015. Do data mining methods matter? : A Wolfcamp "Shale" case study, in: Society of Petroleum Engineers - SPE Hydraulic Fracturing Technology Conference 2015. OnePetro, pp. 136–147. https://doi.org/10.2118/173334-ms

Zhou, Q., Dilmore, R., Kleit, A., Wang, J.Y., 2014. Evaluating gas production performances in marcellus using data mining technologies. J Nat Gas Sci Eng 20, 109–120. https://doi.org/10.1016/j.jngse.2014.06.014

Zhou, Y., 2017. THE APPLICATION OF PRINCIPAL COMPONENT ANALYSIS IN PRODUCTION FORECASTING. URL https://oaktrust.library.tamu.edu/bitstream/handle/1969.1/169590/ZHOU-THESIS-2017.pdf (accessed 8.8.21).

Zhou, Z.H., 2012. Ensemble methods: Foundations and algorithms. Ensemble Methods: Foundations and Algorithms 1–218. https://doi.org/10.1201/B12207/ENSEMBLE-METHODS-ZHI-HUA-ZHOU
